# Single low dose primaquine to reduce gametocyte carriage and *Plasmodium falciparum* transmission after artemether-lumefantrine in children with asymptomatic infection: a randomised, double-blind, placebo-controlled trial

**DOI:** 10.1186/s12916-016-0581-y

**Published:** 2016-03-08

**Authors:** Bronner P. Gonçalves, Alfred B. Tiono, Alphonse Ouédraogo, Wamdaogo M. Guelbéogo, John Bradley, Issa Nebie, Débé Siaka, Kjerstin Lanke, Alice C. Eziefula, Amidou Diarra, Helmi Pett, Edith C. Bougouma, Sodiomon B. Sirima, Chris Drakeley, Teun Bousema

**Affiliations:** Department of Immunology and Infection, London School of Hygiene & Tropical Medicine, London, UK; Department of Biomedical Sciences, Centre National de Recherche et de Formation sur le Paludisme, Ouagadougou, Burkina Faso; MRC Tropical Epidemiology Group, Department of Infectious Disease Epidemiology, London School of Hygiene & Tropical Medicine, London, UK; Department of Medical Microbiology, Radboud University Medical Center, Nijmegen, The Netherlands

**Keywords:** Malaria, Transmission, Gametocytes, Primaquine, Artemisinin-based combination therapies

## Abstract

**Background:**

A single low dose (0.25 mg/kg) of primaquine is recommended as a gametocytocide in combination with artemisinin-based combination therapies for *Plasmodium falciparum* but its effect on post-treatment gametocyte circulation and infectiousness to mosquitoes has not been quantified.

**Methods:**

In this randomised, double-blind, placebo-controlled trial, 360 asymptomatic parasitaemic children aged 2-15 years were enrolled and assigned to receive: artemether-lumefantrine (AL) and a dose of placebo; AL and a 0.25 mg/kg primaquine dose; or AL and a 0.40 mg/kg primaquine dose. On days 0, 2, 3, 7, 10 and 14, gametocytes were detected and quantified by microscopy, Pfs25 mRNA quantitative nucleic acid sequence based amplification (QT-NASBA), and quantitative reverse-transcriptase PCR (qRT-PCR). For a subset of participants, pre- and post-treatment infectiousness was assessed by mosquito feeding assays on days -1, 3, 7, 10 and 14.

**Results:**

Both primaquine arms had lower gametocyte prevalences after day 3 compared to the placebo arm, regardless of gametocyte detection method. The mean (95 % confidence interval) number of days to gametocyte clearance in children with patent gametocytes on day 0 (N = 150) was 19.7 (14.6 – 24.8), 7.7 (6.3 – 9.1) and 8.2 (6.7 – 9.6) for the AL-placebo, the 0.25 mg/kg primaquine dose and the 0.40 mg/kg primaquine dose arms, respectively. While 38.0 % (30/79) of selected gametocytaemic individuals were infectious before treatment, only 1/251 participant, from the AL-placebo group, infected mosquitoes after treatment.

**Conclusions:**

We observed similar gametocyte clearance rates after 0.25 and 0.40 mg/kg primaquine doses. Infectivity to mosquitoes after AL was very low and absent in primaquine arms.

**ClinicalTrials.gov Registration:**

NCT01935882

**Electronic supplementary material:**

The online version of this article (doi:10.1186/s12916-016-0581-y) contains supplementary material, which is available to authorized users.

## Background

Infectiousness to malaria vectors depends on the presence of sexual stage parasites, gametocytes, in the peripheral blood and is essential to sustain transmission in endemic areas [1]. Shortening the duration of gametocyte circulation reduces the probability of parasite spread and might, therefore, facilitate control. Artemisinin-based combination therapy (ACT) is universally adopted as first-line treatment for clinical falciparum malaria [2], effectively clears asexual parasites and immature gametocytes, and is associated with lower post-treatment infectivity based on membrane feeding assays compared to other antimalarials [3]. Mature gametocytes, however, persist after ACT in microscopic or submicroscopic concentrations and residual transmission has been reported following sulphadoxine-pyrimethamine in combination with artesunate [4, 5], dihydroartemisinin-piperaquine [4, 6] and artemether-lumefantrine (AL) [4, 6].

Primaquine, tafenoquine and methylene blue radically clear mature gametocytes with primaquine being the only one commonly used as antimalarial [7]. The World Health Organization recommends a single low dose (0.25 mg/kg) as a gametocytocide in combination with ACT for *Plasmodium falciparum* malaria in elimination and artemisinin resistance containment scenarios [8]. However, wide-scale adoption of this recommendation is limited by safety concerns [9], despite evidence that a higher dose, 0.40 mg/kg, is associated with only minor haematological changes in G6PD normal children [10]. Furthermore, the effect of the 0.25 mg/kg dose in clearing gametocytes or preventing transmission after currently used ACT regimens has not been formally assessed. A dose-finding study in Uganda suggested a 0.40 mg/kg dose is as efficacious in clearing gametocytes as the previously recommended dose of 0.75 mg/kg [10], which has been associated with haemolysis [11]. At present, only mosquito feeding assays can truly determine the lowest efficacious dose of primaquine for preventing transmission [12].

Here, we compare gametocyte dynamics after AL alone or in combination with 0.25 mg/kg or 0.40 mg/kg primaquine dose in children with asymptomatic falciparum infection. We selected asymptomatic parasite carriers since these contribute considerably to the infectious reservoir for malaria [13, 14] and malaria elimination and artemisinin resistance containment strategies will need to involve the targeting of asymptomatic malaria infections [15]. For a subset of participants, post-treatment infectiousness was determined by mosquito membrane feeding experiments.

## Methods

### Role of the funding source

Authors had full access to the trial data. Funders had no role in study design, data collection, analysis, interpretation and decision to publish study findings.

### Study design

For this randomised, double-blind, placebo-controlled trial, participants were recruited between September 2013 and October 2014 from Balonghin, district of Saponé, an area with seasonal malaria in Burkina Faso [16].

**Fig. 1:**
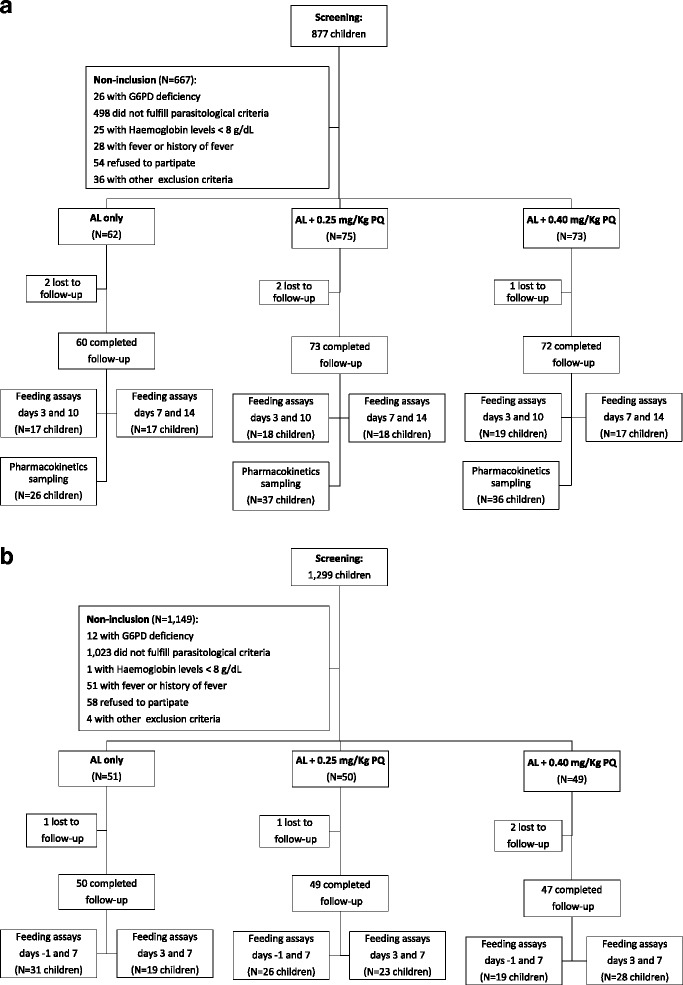
Clinical trial profile. **a** Study Phase where the presence of asexual parasites (1,000 - 200,000 parasites/μL) at screening was an inclusion criterion (initial 143 and final 67 participants). **b** Study Phase where children with patent gametocytes, regardless of their asexual parasite count, at screening were eligible (150 participants). Participants were considered to have a complete follow-up, if they had a total of seven follow-up visits (days 0, 1, 2, 3, 7, 10, 14). *AL* artemether-lumefantrine, *PQ* primaquine

Eligible individuals were asymptomatic children aged 2 to 15 years, weighing 10 kilos or more, with normal glucose-6-phosphate dehydrogenase (G6PD) activity based on BinaxNOW rapid diagnostic test (Alere Inc., Waltham, MA, USA) and carrying patent *P. falciparum* asexual parasites or gametocytes. Initially, individuals were recruited regardless of microscopy gametocyte status (Study Phase A). Since no mosquito infections were detected (0/106 participants), the parasitological criterion for inclusion in infectiousness assessments was modified to include only children with patent gametocytes at screening, regardless of their asexual parasite count, to maximize pre-treatment infectivity (Study Phase B). Exclusion criteria were: haemoglobin level at screening lower than 8 g/dL, fever or history of fever in the last 24 hours, evidence of severe illness, known allergy to study medications, antimalarials taken in the last 48 hours, primaquine use in the last four weeks, blood transfusion in the last 90 days, and non-falciparum malaria infection at screening. Overall, 360 children were enrolled: 210 in Study Phase A (inclusion criterion: asexual parasite density 1,000 - 200,000 parasites/μL) and 150 participants in Study Phase B (inclusion criterion: presence of gametocytes by microscopy) (Fig. 1).

### Ethics, consent and permissions

This clinical trial was conducted in accordance with Good Clinical Practice guidelines, and was registered with ClinicalTrials.gov (number NCT01935882). Ethical clearance was obtained from the London School of Hygiene and Tropical Medicine ethics committee (reference number 6274), and the Comité d’Ethique pour la Recherche en Santé (Ministère de la Santé du Burkina Faso; reference number 2012-10-78). Written informed consent was obtained from parents or guardians, and assent, from children 12-years old or older. The original protocol for the clinical trial (Additional file 1) and the supporting CONSORT checklist (Additional file 2) are provided as supporting information.

### Randomisation and masking

Children were enrolled by the study clinician and subsequently randomly allocated by the study pharmacist, with equal probabilities, to three treatment arms: (1) AL as standard six-dose regimen in combination with a single dose of placebo; (2) AL and a single 0.25 mg/kg primaquine dose; and (3) AL and a single 0.40 mg/kg primaquine dose.

Treatment assignment was stratified by gender, and sealed envelopes in block sizes of six (three study arms and two membrane feeding assays schedules [days 3 and 10 or 7 and 14 after treatment initiation]) were used. After the first 143 children were recruited, mosquito feeding experiments were amended: gametocytaemic children were randomised to have feeding assays on days -1 (one day before treatment initiation) and 7 or 3 and 7.

Treatment concealment was achieved by the addition of a syrup, which masked the colour and taste of primaquine and placebo. Participants, investigators, and staff who were not involved in study drug administration were blinded to study arm allocation.

### Procedures

AL (Coartem®; Novartis Pharma, Basel, Switzerland) was administered as half a tablet (20 mg of artemether and 120 mg of lumefantrine) per 5 kg of body weight in a six-dose regimen over three days. Primaquine doses were prepared as previously described [10] and given with the fifth dose (day 2) of AL. All treatment doses were administered under supervision and with fatty food (biscuits) to ensure adequate absorption [17] and minimise the risk of gastro-intestinal side effects. If a child vomited within 30 minutes after a study medication was given, the treatment was re-administered.

Participants were asked to return to the study clinic on days 0, 1, 2, 3, 7, 10 and 14. On day 0 (day of enrolment), AL administration was initiated. On each follow-up day, study subjects were examined and, except on day 1, had a blood slide prepared to detect malaria parasites and their haemoglobin levels quantified by Hemocue photometer (HemoCue AB, Angelholm, Sweden). Smears were screened for asexual stage parasites and gametocytes, and double-read. Biochemistry and full blood count assessments were performed on days 0, 3 and 7 on venous samples.

Finger-prick-blood samples (50 μL) were collected on all visits, except on day 1, stored in 250 μL of RNAprotect® cell reagent (Qiagen), and used for gametocyte detection and quantification: quantitative nucleic acid sequence based amplification (QT-NASBA) [18] was performed for samples collected on days 0 and 7; and quantitative reverse-transcriptase PCR (qRT-PCR), which allows better quantification [19], was used to estimate gametocyte densities in all available samples for individuals with patent gametocytes on day 0. Briefly, total nucleic acids (NA) were extracted using a MagNAPure LC automatic extractor (Total Nucleic Acid Isolation Kit – High Performance, Roche Applied Science). QT-NASBA was performed directly on total NA samples as described [18], with a minor change in the KCl concentration used: 60 instead of 80 mM. For gametocyte quantification by qRT-PCR, the remaining genomic human and parasite DNA were first removed with the RQ1 DNaseI Digest Kit (Promega), then cDNA was synthesized with the High Capacity cDNA Reverse Transcription Kit (Applied Biosystems); from cDNA samples, the Pfs25 sequence was amplified using primers described by Wampfler et al [19], GoTaq® qPCR Master Mix (Promega), and CFX96™ Real-Time PCR Detection System (BIO-RAD), using the standard cycling program as described in the manufacturer’s instructions. Amplification was set to continue for 40 cycles and the cut-off for positivity was set at assigned gametocyte density of more than 0.02 gametocytes per μL, determined by a dilution series of mature stage V in vitro produced gametocytes (NF54 strain).

A subset of recruited children was invited to participate in membrane feeding assays [20] at two time-points (Fig. 1), with the number of participants being dictated by the availability of mosquitoes. For each experiment, venous blood was taken and offered to 60 or more locally-reared female *Anopheles gambiae* mosquitoes. Fully fed mosquitoes were selected, kept on glucose at 27–29 °C and dissected seven days later. Midguts were examined for the presence of oocysts.

### Primary and secondary endpoints

The primary efficacy endpoint was gametocyte clearance time (i.e. number of days to undetectable gametocyte levels) in the 0.25 mg/kg primaquine arm compared to the 0.40 mg/kg primaquine arm. The primary safety endpoint was maximal fall in haemoglobin levels during follow-up. Secondary efficacy endpoints were: gametocyte prevalence on days 3, 7, 10 and 14, and proportion of mosquitoes developing infection and their oocyst counts in feeding assays performed after treatment administration. Secondary safety endpoints included: number of participants requiring blood transfusion, maximal percentage decrease in haemoglobin concentration, proportion of participants with haemoglobin levels below 5 g/dL and number of serious adverse events.

### Sample size

Sample size calculations were based on non-inferiority of 0.25 mg/kg versus 0.40 mg/kg primaquine dose. For the gametocyte clearance endpoint, the non-inferiority margin was 2.5 days [21]. If mean clearance time was 6.3 days [10] in both arms, with a standard deviation of 3, then 40 subjects per arm would give over 90 % power at the one sided 0.025 level. For QT-NASBA-determined gametocyte prevalence on day 7, the non-inferiority margin was 12 %. If prevalence in both arms was 10.6 % [10], then 120 subjects per arm would give over 80 % power at the one sided 0.025 level. The number of feeding experiments and mosquitoes used in each assay were limited by logistical considerations and were not based on sample size calculations.

### Statistical analysis

Stata 12.0 (Stata Corporation, College Station, TX, USA) and SAS 9.3 (SAS Institute, Cary, NC, USA) were used for statistical analysis. The mean time to gametocyte clearance was estimated for each treatment arm using a non-linear model [21] (Additional file 3); qRT-PCR data from children with patent gametocytes on day 0 were used (N = 150). For the 0.25 mg/kg primaquine dose, a 95 % confidence interval (CI) for the difference with the 0.40 mg/kg dose was calculated. The non-linear model was also used to assess superiority of the primaquine arms over the placebo group. Gametocyte prevalences on different visit days were compared between study arms by χ^2^ or Fisher’s exact test. Non-parametric tests (Mann-Whitney and Kruskal-Wallis) were used to compare parasite levels among different study groups. To quantify the effect of primaquine on transmission, post-treatment mosquito infection rates were compared between treatment arms. The maximal fall in haemoglobin levels measured by Hemocue photometer was presented as mean (95 % CI) per study arm; Student’s t-test was used for pair-wise comparisons.

## Results

We screened 2,176 (877 in Study Phase A and 1,299 in Study Phase B) and enrolled 360 children (Fig. 1). Twenty participants were enrolled despite having < 1,000 asexual parasites/μL and therefore not fulfilling all enrolment criteria but were retained in the analysis (Additional file 3). The median (interquartile range [IQR]) age at enrolment was eight (five to ten) years (Table 1). Median (IQR) asexual stage parasites concentration at enrolment was 1,824.5 (520–5,041) parasites/μL and was similar among individuals receiving different treatment regimens.

**Table 1:** Study population

	AL only	AL + 0.25 mg/kg PQ	AL + 0.40 mg/kg PQ
Asymptomatic parasite carriers (Study Phase A)			
Female (%)	51.6	52.0	53.4
Age (years)	7.5 (4 – 10)	8 (5 – 10)	8 (4 – 10)
Haemoglobin (g/dL) at enrolment^a^	10.8 (10.2 – 11.4)	11.3 (10.7 – 11.9)	11.7 (11.2 – 12.2)
Asexual parasite levels at enrolment (parasites/μL; Microscopy)	2,972.5 (1,147 – 6,865)	2,503 (939 – 5,798)	3,339 (874 – 7,358)
Gametocyte prevalence (%) at enrolment (Microscopy)	17.7	32.0	20.5
Gametocyte prevalence (%) at enrolment (QT-NASBA)	96.4	92.9	85.5
Asymptomatic gametocyte carriers (Study Phase B)			
Female (%)	43.1	46.0	42.9
Age (years)	8 (5 – 11)	7 (6 – 11)	8 (6 – 11)
Haemoglobin (g/dL) at enrolment^a^	11.4 (11.1 – 11.8)	11.7 (11.4 – 12.1)	11.5 (11.1 – 11.9)
Asexual parasite levels at enrolment (parasites/μL; Microscopy)	488 (226 – 1,670)	957 (248 – 3,004)	908.5 (198 – 2,297)
Gametocyte prevalence (%) at screening (Microscopy)	100	100	100
Gametocyte prevalence (%) at enrolment (Microscopy)	69.4	55.3	83.3
Gametocyte prevalence (%) at enrolment (QT-NASBA)	95.9	93.7	100.0
Gametocyte levels at enrolment (parasites/μL)^b^	17.3 (7.2 - 45.1)	18.6 (7.8 - 75.3)	16.6 (7.2 - 40.0)

Means and 95 % confidence intervals are presented for haemoglobin levels at enrolment; medians and interquartile ranges are presented for parasite levels (asexual or sexual stages) and age. Study Phase A = presence of asexual parasites (1,000 - 200,000 parasites/μl) at screening as an inclusion criterion; Study Phase B = presence of patent gametocytes at screening as an inclusion criterion

*AL* artemether-lumefantrine, *PQ* primaquine

^a^Measured by Hemocue photometer; similar values observed with full blood count assessment using venous samples

^b^Quantified by qRT-PCR (only children with patent gametocytes on day 0 [N = 150] were included [100/150 were enrolled during Study Phase B]; see Methods)

Gametocyte prevalence determined by microscopy was not significantly different between study arms at enrolment, although during Study Phase A it was higher in the 0.25 mg/kg primaquine arm (32.0 %) compared to the AL-placebo and the 0.40 mg/kg primaquine arms (17.7 and 20.5 %, respectively). Both primaquine arms had lower microscopically-detectable gametocyte prevalence on days 7, 10 and 14, but not on days 2 and 3, compared to the AL-placebo group (Table 2). Similar results were obtained when only children with gametocytes on day 0 were included in this comparison (Additional file 3: Table S1).

**Table 2:** Gametocyte carriage during follow-up

Study Phase A (asymptomatic parasite carriers)	AL	AL + 0.25 mg/kg PQ	AL + 0.40 mg/kg PQ	P-values	P-values
	% (n/N)	% (n/N)	% (n/N)	(AL vs.AL + 0.25 mg/kg PQ)	(AL vs.AL + 0.40 mg/kg PQ)
Gametocyte prevalence by microscopy					
Day 0	17.7 (11/62)	32.0 (24/75)	20.5 (15/73)	0.08	0.83
Day 2	12.9 (8/62)	16.4 (12/73)	13.7 (10/73)	0.63	1.00
Day 3	9.8 (6/61)	22.9 (16/70)	14.3 (10/70)	0.06	0.59
Day 7	6.7 (4/60)	4.2 (3/72)	0.0 (0/71)	0.70	0.04
Day 10	3.3 (2/60)	1.4 (1/71)	0.0 (0/71)	0.60	0.21
Day 14	0.0 (0/58)	0.0 (0/73)	0.0 (0/71)	-	-
Gametocyte prevalence by Pfs25 QT-NASBA					
Day 0	96.4 (54/56)	92.9 (65/70)	85.5 (53/62)	0.46	0.06
Day 7	46.3 (25/54)	20.3 (14/69)	16.4 (11/67)	0.003	0.001
Study Phase B (patent gametocyte carriers at screening)	AL	AL + 0.25 mg/kg PQ	AL + 0.40 mg/kg PQ	P-values	P-values
	% (n/N)	% (n/N)	% (n/N)	(AL vs.AL + 0.25 mg/kg PQ)	(AL vs.AL + 0.40 mg/kg PQ)
Gametocyte prevalence by microscopy					
Day 0	69.4 (34/49)	55.3 (26/47)	83.3 (40/48)	0.21	0.15
Day 2	34.7 (17/49)	20.4 (9/44)	28.9 (13/45)	0.17	0.66
Day 3	28.6 (14/49)	10.9 (5/46)	15.6 (7/45)	0.04	0.15
Day 7	20.4 (10/49)	6.4 (3/47)	6.7 (3/45)	0.07	0.07
Day 10	17.4 (8/46)	4.3 (2/47)	0.0 (0/44)	0.05	0.006
Day 14	13.6 (6/44)	0.0 (0/40)	0.0 (0/38)	0.03	0.03
Gametocyte prevalence by Pfs25 QT-NASBA					
Day 0	95.9 (47/49)	93.7 (45/48)	100.0 (46/46)	0.68	0.50
Day 7	55.1 (27/49)	20.4 (10/49)	17.0 (8/47)	0.001	<0.001
Only patent gametocyte carriers at enrolment	AL	AL + 0.25 mg/kg PQ	AL + 0.40 mg/kg PQ	P-values	P-values
	% (n/N)	% (n/N)	% (n/N)	(AL vs.AL + 0.25 mg/kg PQ)	(AL vs.AL + 0.40 mg/kg PQ)
Gametocyte prevalence by Pfs25 qRT-PCR					
Day 0	95.6 (43/45)	98.0 (48/49)	98.0 (49/50)	0.60	0.60
Day 2	93.2 (41/44)	87.5 (42/48)	90.4 (47/52)	0.49	0.72
Day 3	88.4 (38/43)	75.5 (37/49)	92.2 (48/52)	0.18	0.72
Day 7	66.7 (28/42)	34.0 (17/50)	27.8 (15/54)	0.003	<0.001
Day 10	65.8 (27/41)	28.6 (14/49)	20.7 (11/53)	0.001	<0.001
Day 14	42.5 (17/40)	10.2 (5/49)	16.7 (9/54)	0.001	0.01
	Mean (95 % CI)	Mean (95 % CI)	Mean (95 % CI)		
AUC gametocyte densities over time (in gametocytes μL^-1^ days)^b^	14.7 (5.5 – 24.0)	5.5 (1.1 – 10.0)	5.6 (2.1 – 9.1)	-	-
Gametocyte clearance time (in days)	19.7 (14.6 – 24.8)	7.7 (6.3 – 9.1)	8.2 (6.7 – 9.6)	-	-
				P-values	P-values
	AL vs. AL + 0.25 mg/kg PQ	AL vs. AL + 0.40 mg/kg PQ	AL + 0.25 mg/kg PQ vs. AL + 0.40 mg/kg PQ	(AL vs.AL + 0.25 mg/kg PQ)	(AL vs.AL + 0.40 mg/kg PQ)
Difference in gametocyte clearance time (in days)	12.0 (6.7 – 17.3)	11.5 (6.2 – 16.8)	-0.5 (- 2.5 – 1.6)	<0.001	<0.001

Gametocyte prevalences, clearance times and area under the curve (AUC) of gametocyte distributions over time are presented. Gametocyte clearance time: the rate with which malaria sexual stage parasites were cleared was estimated for children with patent gametocytes on day 0; qRT-PCR data were used (N = 150; see Additional file 3). Gametocyte prevalences were similar in primaquine study arms throughout the follow-up (all P-values > 0.05), except on day 0 (microscopy-based detection; P = 0.004) during Study Phase B

*AL* artemether-lumefantrine, *PQ* primaquine

^a^50 children from Study Phase A and 100 from Study phase B

^b^Only qRT-PCR measurements on days 3, 7, 10 and 14 for children with qRT-PCR data on all these days are included

### Molecular detection of gametocytes

Gametocyte prevalences determined by Pfs25 mRNA QT-NASBA were considerably higher than by microscopy (Table 2). On day 7, QT-NASBA-determined gametocyte prevalence was 50.5 % in the AL-placebo arm, 20.3 % in the 0.25 mg/kg primaquine arm (P < 0.001, versus control) and 16.7 % in the 0.40 mg/kg primaquine arm (P < 0.001, versus control; P = 0.50, versus 0.25 mg/kg primaquine dose).

**Fig. 2:**
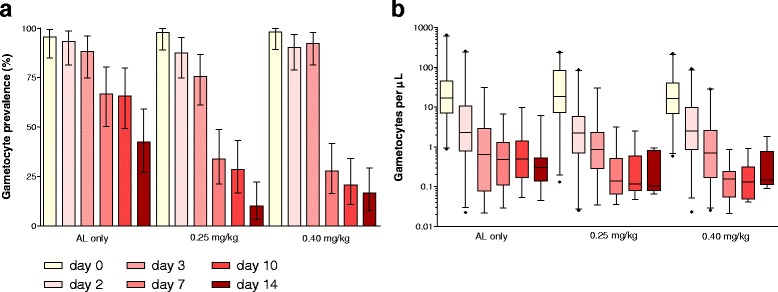
Gametocyte prevalences (**a**) and densities (**b**) measured by qRT-PCR in children with patent gametocytes on day 0. 95 % confidence intervals are presented in (**a**). Samples were considered to be gametocyte negative if assigned levels were lower than 0.02 gametocytes per μL. AL = artemether-lumefantrine; 0.25 mg/kg = 0.25 mg/kg primaquine arm; 0.40 mg/kg = 0.40 mg/kg primaquine arm

Gametocyte prevalences and densities measured by qRT-PCR for children with microscopically detectable gametocytes on day 0 are presented in Fig. 2. Gametocyte levels in gametocyte-positive visits did not differ between treatment arms on days 0, 2, 3 and 14; on days 7 and 10, the 0.40 mg/kg primaquine arm had lower gametocyte levels compared to the placebo group (Additional file 3: Table S2). Gametocyte densities were similar in primaquine arms throughout the follow-up, as gametocyte clearance rates. Children receiving either dose carried sexual stage parasites for a shorter time compared to the AL only arm (19.7 [14.6 – 24.8], 7.7 [6.3 – 9.1] and 8.2 [6.7 – 9.6] days [mean and 95 % CI] to gametocyte clearance for the AL-placebo, the 0.25 mg/kg primaquine and the 0.40 mg/kg primaquine arms, respectively; Table 2).

### Infectiousness to mosquitoes

In Study Phase A, when individuals were recruited regardless of gametocytaemia by microscopy, 106 children participated in membrane feeding experiments on days 3 (N = 54), 7 (N = 52), 10 (N = 54) and 14 (N = 52); during this Study Phase, no mosquitoes developed infection (Table 3). In Study Phase B, 149 individuals with patent gametocytes at screening participated in feeding experiments prior to treatment (N = 79) and on days 3 (N = 70; 19, 23 and 28 in the AL-placebo, the 0.25 mg/kg primaquine and the 0.40 mg/kg primaquine arms, respectively) and 7 (N = 144; 49, 49 and 46 in the AL-placebo, the 0.25 mg/kg primaquine and the 0.40 mg/kg primaquine arms, respectively). On average, 45.3 (range 25 - 94) mosquitoes were dissected per assay. Of the experiments conducted prior to treatment, 38.0 % (30/79) resulted in ≥ 1 infected mosquito. In these 30 infectious feeds, the median proportion of infected mosquitoes was 20.0 % (IQR 8.0 – 39.0) and median oocyst count per infected mosquito was 5 (IQR 2 – 15). Only one individual infected mosquitoes following treatment, in the AL only arm and infected 4/46 mosquitoes on day 7; this participant, who was also infectious before treatment administration (18/48 infected mosquitoes), had submicroscopic gametocytaemia at the time of feeding on day 7.

**Table 3:** Mosquito feeding assays

	AL only	AL + 0.25 mg/kg PQ	AL + 0.40 mg/kg PQ
Study phase A			
Number of mosquitoes infected/dissected (proportion)			
Follow-up day			
3	0/755 (0)	0/816 (0)	0/862 (0)
7	0/781 (0)	0/777 (0)	0/767 (0)
10	0/822 (0)	0/848 (0)	0/890 (0)
14	0/855 (0)	0/890 (0)	0/841 (0)
Study phase B			
Number of mosquitoes infected/dissected (proportion)			
Follow-up day			
One day before treatment	207/1402 (0.15)	54/1195 (0.05)	80/897 (0.09)
3	0/863 (0)	0/1053 (0)	0/1281 (0)
7	4/2104 (0.002)	0/2179 (0)	0/2016 (0)

*AL* artemether-lumefantrine; *PQ* primaquine

### Adverse events and haematological changes in G6PD normal children

A total of 57 mild and moderate adverse events were recorded; eight (seven mild, one moderate), occurring in three, one and four individuals assigned to the AL-placebo, the 0.25 mg/kg primaquine and the 0.40 mg/kg primaquine arms, respectively, were considered to be possibly related to study participation (Additional file 3: Table S3). For five children who vomited on days 0 or 1, study drug was re-administered. No serious adverse events were observed.

**Fig. 3:**
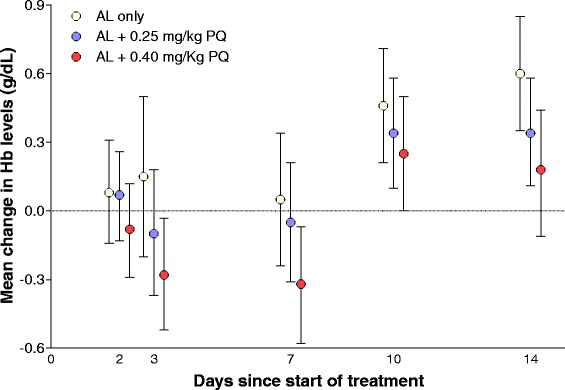
Changes in haemoglobin levels from baseline values. Means and 95 % confidence intervals are presented. Haemoglobin levels determined by Hemocue photometer on day 0 were used as baseline measurement; whenever haemoglobin concentrations on day 0 were not available or were only quantified by full blood count analysis on venous samples, Hemocue results at screening were used: the median (IQR) interval between screening and the day 0 of follow-up was 2 (1 – 2) days. *AL* artemether-lumefantrine, *PQ* primaquine, *Hb* haemoglobin, *IQR* interquartile range

At enrolment, haemoglobin levels did not differ between treatment arms (Table 1). One child had 6 g/dL on day 0, but this was most likely due to measurement error: on day 2, haemoglobin concentration was 10.9 g/dL. Median (IQR) uncorrected reticulocyte percentage at enrolment was 2.0 % (1.5 – 2.6). The average maximal fall in haemoglobin levels for children with measurements on all visits (227/360) was larger (P = 0.006), in absolute value, in the 0.40 mg/kg primaquine arm, -1.21 (95 % CI, -1.45 to -0.97, N = 76) g/dL, compared to the placebo arm, -0.71 (95 % CI, -0.98 to -0.44, N = 72). The haemoglobin drop for participants receiving the 0.25 mg/kg primaquine dose was -0.96 (95 % CI, -1.18 to -0.73, N = 79; P = 0.16 and 0.12, versus control and 0.40 mg/kg primaquine arms, respectively). Similar results were obtained when considering maximal percentage drop (Additional file 3: Table S4). The lowest haemoglobin values relative to baseline were observed on days 3 and 7 (Fig. 3). A total of 35 children (9/113 [8.0 %], 12/125 [9.6 %] and 14/122 [11.5 %] in the AL-placebo, the 0.25 mg/kg primaquine and the 0.40 mg/kg primaquine arms, respectively) had a haemoglobin drop of 2 or more g/dL (Additional file 3: Table S5): in 2, one in each primaquine arm, haemoglobin fell more than 4 g/dL and levels recovered before follow-up was completed. On days 1 and 2, three children reported haemoglobinuria. No child had haemoglobin levels < 5 g/dL, and no blood transfusions were required. Renal and liver laboratory abnormalities were detected in four and seven children, respectively (Additional file 3: Table S6).

## Discussion

Since 2012, the WHO recommends a single 0.25 mg/kg primaquine dose as gametocytocide but this dosage had never been formally assessed for gametocyte clearance after ACT. In this study, we recruited asymptomatic parasitaemic children, a group that is believed to contribute significantly to transmission in endemic areas [13] and would potentially be given low dose primaquine if used in future community chemotherapy campaigns. We showed that individuals receiving a 0.25 mg/kg primaquine dose in combination with AL had similar gametocyte clearance time compared to children in the 0.40 mg/kg primaquine arm. Both regimens were associated with significantly shorter post-treatment gametocyte circulation compared to the ACT partner drug alone. In the subset of participants who had their infectivity quantified by feeding experiments, infectiousness was effectively reduced after treatment in all arms. Our observations suggest that (1), on the basis of gametocyte carriage, the primaquine dose of 0.25 mg/kg is as effective as the previously evaluated dose, 0.40 mg/kg; and (2) gametocytaemia is not a good predictor of post-treatment infectivity [12]: infectivity is very low after AL and not detected after AL-primaquine.

Previous trials on low dose primaquine’s transmission-reducing properties have assessed efficacy based on gametocyte carriage [10]. However, the non-linear relationship between gametocyte levels and mosquito infection rates [22] during experimental infections suggests that reductions in numbers of circulating gametocytes might not be directly translated into changes in transmission potential. Additionally, primaquine might have an impact on infectiousness before sexual stage parasites clearance is observed [12]. Pfs25 mRNA transcripts are more abundantly present in female gametocytes [23] and may also be detected in non-infectious gametocytes [24]. It was recently hypothesized that male gametocytes might be more sensitive to 8-aminoquinolines in vivo [25] and, since they represent a smaller fraction of the total gametocyte population compared to female gametocytes [26], their clearance will only minimally influence gametocytaemia by Pfs25 mRNA-based quantification methods but significantly affect infectiousness [27]. Thus, estimating the effect of primaquine on transmission requires direct assessment of infectivity. In this trial, we quantified infectiousness for 255 individuals and 22,894 dissected mosquitoes and adapted our study design to maximise discriminative power by enriching our study population for individuals with microscopically-detectable gametocytes.

We observed that 38.0 % of gametocytaemic individuals infected mosquitoes before treatment. This is within the normal range of infectivity in patent gametocyte carriers [28], although considerably higher infection rates have been reported [22, 29]. After treatment, only one child infected mosquitoes, suggesting that transmissibility was substantially reduced by AL [3]. Low infectivity after AL alone made it impossible to detect an added value of primaquine in this context. The almost complete absence of infected mosquitoes post-treatment contrasts with the limited efficacy of artemisinin derivatives against mature gametocytes, and could be related to lumefantrine, that inhibits male gametocyte exflagellation and reduces oocyst numbers in vitro [30]. Factors intrinsic to these infections (e.g. gametocyte sex ratios) or to the host (e.g. transmission-reducing immunity) could also have contributed to this low infectiousness. Another explanation is that membrane feeding experiments, which in general underestimate infectiousness compared to skin feeding assays [28], might not have been sensitive enough to detect very low infectivity. The successful detection of pre-treatment infectiousness, however, suggests that low sensitivity cannot fully explain our findings. Only a few studies (summarised in Additional file 3: Table S7) assessed the effect of AL on transmission: similar to our observations, in The Gambia, post-treatment gametocyte carriage was observed, but mosquito infections were not detected [3]. In contrast, in Kenya, transmission was observed after AL [6] and clonally complex infections were detected by PCR in infected mosquito guts.

Determining the true impact of primaquine on malaria infectivity should be high on the malaria elimination agenda. Larger clinical studies with appropriate sample sizes to test efficacy outcomes involving infectiousness reduction are necessary. These studies may also determine the optimum timing of primaquine administration. In our study, primaquine was administered on the last day of AL use, to follow the same treatment procedures as a previous dose-finding trial in Uganda, although there are logistical advantages to administering primaquine with the first supervised dose of AL. Ideally, these studies will include symptomatic and asymptomatic malaria infections to demonstrate the efficacy of primaquine in all infections that contribute to the human infectious reservoir for malaria. These studies will be logistically complex and need to consider all factors that influence infectiousness as they might also influence efficacy estimates: (1) membrane feeding assays underestimate infectivity compared to direct skin feeding assays and consequently low level post-treatment infectivity might be misclassified as non-infectiousness when using this artificial system; (2) the number of mosquitoes used in each assay might not represent the number of mosquito bites an infectious individual would receive in natural settings (especially acknowledging as this is a single time point assessment), and binary outcomes (e.g. “infectious” versus “not-infectious”) might be less relevant than within-individual changes in mosquito infection rates; (3) baseline infectivity will depend on the inclusion criteria and although recruiting individuals with high gametocyte densities would maximize the power to observe changes in infectiousness with treatment, these individuals might not be representative of an “average” infected person. As duration and intensity of infectiousness are likely to depend on host age and transmission setting, individuals from different ages and study sites, representing a wide spectrum of endemicities, would need to be recruited.

Primaquine-induced haemolysis in individuals with G6PD deficiency is a concern for control programs and one major reason why this drug is not widely used in Africa. Here, a rapid diagnostic test was used to exclude individuals with this condition. No cases of severe haemolysis were observed, corroborating previous observations made in symptomatic children with normal G6PD activity receiving a single 0.40 mg/kg primaquine dose [10]. However, contrary to this previous trial undertaken in Uganda, children receiving 0.40 mg/kg primaquine in this study had larger haemoglobin drops compared to the placebo group, suggesting that mild haemolysis might have occurred. While our study provides detailed haematological assessment after primaquine administration in G6PD normal non-anaemic children with asymptomatic falciparum infections, G6PD deficiency could lead to greater haemoglobin drops and studies recruiting G6PD deficient individuals are necessary.

## Conclusions

The ultimate goal of using primaquine in falciparum malaria in conjunction with ACT is to minimise post-treatment malaria transmission, reduce the transmission of artemisinin-resistant parasites and accelerate attempts to eliminate malaria [31]. Although mathematical models have been developed to estimate the potential impact of this drug on population-level transmission [32], its effect on infectiousness needs to be quantified. In this study, children receiving 0.40 mg/kg or 0.25 mg/kg primaquine doses cleared gametocytes with similar rates; both treatment regimens reduced gametocyte carriage compared to ACT alone. Feeding assays, however, indicate that infectiousness is considerably reduced by AL alone, suggesting that the benefit of adding primaquine to first-line antimalarials is influenced by partner drug.

## Additional files

Additional file 1: Protocol. (PDF 370 kb) Additional file 2: CONSORT checklist. (PDF 89 kb) Additional file 3: Supplementary Appendix. (PDF 102 kb)
