# Supplementary material for: Single low dose primaquine to reduce gametocyte carriage and Plasmodium falciparum transmission after artemether-lumefantrine in children with asymptomatic infection: a randomised, double-blind, placebo-controlled trial
Source: BMC Med. 2016 Mar 8;14:40. doi: 10.1186/s12916-016-0581-y (PMC4782330; doi:10.1186/s12916-016-0581-y)
Supplement: Additional file 1: — Protocol. (PDF 370 kb) [file 12916_2016_581_MOESM1_ESM.pdf]

1. TITLE OF THE PROJECT

Evaluation of the efficacy and safety of low dose primaquine for clearance of gametocytes in asymptomatic individuals infected with *P. falciparum* in Burkina Faso (LOPRIM)

CONTENTS

1. TITLE OF THE PROJECT 1

2. SPONSOR AND REVIEWING ETHICS COMMITTEES 4

3. INVESTIGATORS AND INSTITUTIONAL AFFILIATIONS........ 4

3. ABSTRACT........ 5

4. LIST & DEFINITIONS OF ABBREVIATIONS, ACRONYMS 6

5. INTRODUCTION/BACKGROUND........ 7

5.1 Safety profile of primaquine and G6PD deficiency........ 7

5.2 G6PD deficiency in Burkina Faso........ 8

5.3 Assessing a safe dose of PQ – preliminary data from Uganda........ 9

5.4 Mosquito feeding assays to confirm the efficacy of a lower dose of PQ 10

6. JUSTIFICATION OF THE STUDY 11

7. MAIN OBJECTIVE........ 11

8. SPECIFIC OBJECTIVES AND HYPOTHESES TO BE TESTED........ 11

9. DESIGN AND METHODOLOGY........ 12

9.1. General study design 12

9.2. Study site and population 13

9.3 Participant recruitment and screening 13

9.4. Enrolment 14

9.5. Randomization and blinding 14

9.6. Treatment and administration procedures 15

9.5. Follow-up and samples taken 16

9.6 Parasite detection........ 17

9.7 Pharmacological analysis 18
9.8 Haematology and biochemistry 20
9.9 G6PD deficiency testing 20
9.10 Membrane feeding assays 20
10. SAMPLE SIZE CONSIDERATIONS 22
10.1 Sample size for efficacy 22
10.2 Sample size for safety 22
11. DATA ANALYSIS PLAN 23
12. ETHICAL CONSIDERATIONS 23
12.1 Confidentiality 24
12.2 Participant reimbursement 24
12.3 Good clinical practice 24
13. SAFETY MANAGEMENT 26
13.1 Safety management 26
13.1.1 Withdrawal of individual subjects 26
13.1.2 Premature termination of the study 26
13.2 Management of haemolysis 26
13.2.1 Measures of haemolysis 27
13.3 Safety reporting 29
13.3.1 Adverse events 29
13.3.2 Serious adverse events 29
13.3.3 Adverse Event Data Collection 30
13.3.4 Assessment of causality 31
13.4 Safety follow-up 31
13.4.1 Follow-up of subjects that voluntarily withdraw participation 31
13.4.2 Follow-up of adverse events 32
13.4.3 Follow-up of serious adverse events 32

13.5 Local Safety Monitor and Data Safety Monitoring Board (DSMB) 33

13.6 Data monitoring 33

14. TIME FRAME AND DURATION OF THE PROJECT 34

15. REFERENCES 34

16. ROLES INVESTIGATORS 35

2. SPONSOR AND REVIEWING ETHICS COMMITTEES

Sponsor:

London School of Hygiene and Tropical Medicine

Keppel Street, London WC1E 7HT

Tel +44 (0) 20 7927 2626

Fax +44 (0) 20 75805636

e-mail: patricia.henley@lshtm.ac.uk

Reviewing ethics committees:

- London School of Hygiene and Tropical Medicine
  Keppel Street, London WC1E 7HT
  Tel +44 (0) 20 7927 2626
  Fax +44 (0) 20 75805636
  e-mail: ethics@lshtm.ac.uk

- Comité d'Ethique pour la Recherche en Santé:
  Ministère de la Santé du Burkina Faso
  03 BP: 7009 Ouagadougou 03
  Tel : +22650326284

- Comité Institutionnel de Bioéthique
  Centre National de Recherche et de Formation
  Sur le Paludisme (CNRFP)
  01 BP: 2208 Ouagadougou 01
  Tel : +22650324695

3. INVESTIGATORS AND INSTITUTIONAL AFFILIATIONS

Sodiomon Sirima, MD PhD[1] Local Principal Investigator

André Lin Ouédraogo, PhD[1]

Guido Bastiaens[2]

Chi Eziefula, MD[3]

Alfred Tiono, MD PhD[1]

Teun Bousema, PhD[2,3] International Principal Investigator

Chris Drakeley, PhD[2] International Principal Investigator

[1] Centre National de Recherche et de Formation sur le Paludisme, Ouagadougou, Burkina Faso

[2]London School of Hygiene and Tropical Medicine, London, UK

[3]Radboud University Nijmegen Medical Centre, Nijmegen, the Netherlands

3. ABSTRACT

Primaquine (PQ) is currently the only available drug that can clear mature transmission stages of *P. falciparum* parasites. PQ was previously shown to clear gametocytes that persist after artemisinin-combination therapy. PQ may therefore play a role in malaria elimination campaigns by preventing malaria transmission. However, there are safety concerns about the use of PQ at the currently recommended dose of 0.75mg/kg in individuals who are glucose-6-phosphate dehydrogenase (G6PD) deficient. PQ causes transient but significant haemolysis in G6PD deficient individuals; this side-effect is dose dependent. There are indications that a lower dosing of PQ may effectively reduce gametocyte carriage but the lowest efficacious dose for gametocyte clearance is currently unknown. Moreover, there is no direct evidence on the extent to which (low dose) PQ prevents malaria transmission to mosquitoes.

In the current study we aim to identify the lowest efficacious dose of PQ in individuals with normal G6PD function. Children with asymptomatic malaria, normal haemoglobin levels (>8g/dL) and normal G6PD enzyme function will be randomized to treatment with artemether-lumefantrine alone or in combination with PQ. All enrolled individuals will receive a full three-day course of AL, and will be randomized to receive a dose of primaquine or placebo with their fifth dose of AL. Sampling will be as follows: all individuals have finger prick blood samples on days 0, 2, 3, 7, 10 and 14 for malaria parasites (asexual and sexual), haemoglobin (using Hemocue®) and into an EDTA tube for gametocyte detection by molecular methods. A venipuncture sample will be obtained for membrane feeding assays on two occasions during this study period. For this, participants will be randomly selected to be enrolled in membrane feeding assays on either days 3 and 10 or days 7 and 14.

4. LIST & DEFINITIONS OF ABBREVIATIONS, ACRONYMS

ACT	artemisinin-based combination therapy
AUC	area under the curve
AE	adverse event
AL	artemether-lumefantrine
AS	artesunate
C_{max}	peak plasma drug concentration
CI	confidence interval
CNRFP	Centre National de Recherche et Formation sur le Paludisme
CRF	case record form
D0, D1, D2	day 0, Day 1, Day 2 of study medication administration
DSMB	data safety and monitoring board
EIR	entomological inoculation rate
G6PD	Glucose-6-phosphate dehydrogenase
GCP	good clinical practice
GCT	gametocyte clearance time
GMR	geometric mean ratio
Hb	haemoglobin
IMCI	Integrated Management of Childhood Illness
ITN	insecticide treated net
IRB	institutional review board
HPLC	high performance liquid chromatography
LLIN	long-lasting insecticide treated net
LSHTM	London School of Hygiene and Tropical Medicine, London, UK
PQ	primaquine
PQ1, PQ2	test doses of primaquine
PQ-R	reference dose of primaquine (0.75mg/kg)
QT-NASBA	real-time quantitative nucleic acid sequence-based amplification
RUNMC	Radboud University Nijmegen Medical Centre, Nijmegen, the Netherlands
SAE	serious adverse event

SD	standard deviation
SP	sulphadoxine-pyrimethamine
T_{max}	time to reach peak plasma drug concentration
UCSF	University of California, San Francisco
UMSP	Uganda Malaria Surveillance Project
UNCST	Uganda National Council of Science and Technology
WHO	World Health Organization

5. INTRODUCTION/BACKGROUND

A new global effort is underway to step up malaria control and push towards the elimination of malaria as a public health problem [1]. Some substantial successes have been achieved in shrinking the global distribution of malaria. In Africa, effective elimination programmes have been initiated in Zanzibar and South Africa. There are now 8 African countries with a commitment to malaria elimination. This call for elimination has created a drive for the development of innovative tools to reduce malaria transmission. One such tool is primaquine (PQ). It is a drug which can efficiently block the transmission of *Plasmodium falciparum* malaria from humans to mosquitoes by clearing the parasite's transmission stages, gametocytes that persist after artemisinin-combination therapy (ACT) [2,3]. A study conducted in Tanzania in symptomatic parasitized children demonstrated a dramatic reduction of gametocyte circulation time from 28.6 days with ACT alone to 6.3 days with ACT-PQ [4]. The World Health Organization (WHO) recommends the use of a single dose of PQ as part of malaria elimination programmes: "As the anti-gametocyte effects of artemisinins are incomplete, malaria elimination programmes require that artemisinin-based therapies be combined with primaquine to block transmission more effectively" (from Malaria Control and Elimination 2008, WHO publication).

5.1 Safety profile of primaquine and G6PD deficiency

Given its widespread use over the last fifty years, there is extensive experience with regards the safety and side effects of PQ. The main side effects are as follows:

i) Gastro-intestinal symptoms if not given with food
ii) Methaemoglobinaemia
iii) Transient haemolysis in individuals with a predisposition such as glucose-6 phosphate dehydrogenase (G6PD) deficiency.

The haemolysis is mostly in aged erythrocytes. Therefore, the reticulocytosis (proliferation of young red blood cells) in acute malaria affords some protection, as the population of red ells is relatively younger.

The haemolytic side effect of PQ is dose-related. Haemolysis is more commonly observed after prolonged PQ treatment but has also been observed in African populations following a single dose of PQ [2, 5]. This haemolysis was self-limiting, largely restricted to G6PD deficient individuals and did not lead to clinical symptoms. Nevertheless, any drug-induced haemolysis is reason for concern and additional studies to determine a safer alternative or saver dosing [6].

5.2 G6PD deficiency in Burkina Faso

Haemolysis after PQ is strongly associated with G6PD deficiency. G6PD enzyme function varies widely in different regions as a consequence of different mutations underlying G6PD deficiency. The range of mutant alleles (over 140 have been characterized) result in varying degrees of deficiency of this enzyme. G6PD enzyme deficiency causes a reduction in this enzyme function. This leaves red cells with lower amounts of NADPH (reduced nicotinamide adenine dinucleotide phosphate) with the result that they are susceptible to oxidative stress. Subsequent oxidative stress can lead to haemolysis. Primaquine exposure leads to transient, dose-dependent intravascular haemolysis in individuals carrying the mutant allele. The severity of the haemolysis depends on the degree of enzyme deficiency. The most common G6PD variant in Burkina Faso, and Africa as a whole, is the A-variant [7, 8]. In 352 samples from clinical malaria patients from around Ouagadougou, all G6PD A-mutations involved the A376G/G202A mutation [8].The A- G6PD variant encoded by these mutations has up to 80% enzyme function compared to wild type. This variant is associated with mild haemolysis in the presence of stimuli such as PQ. By contrast, some Southeast Asian and Mediterranean variants code for severe deficiency, and PQ may provoke a severe haemolysis in G6PD deficient individuals.

Table 1. G6PD variants, geography and broad risk of haemolysis

G6PD variant	Geographic region	Risk/ severity of haemolysis
B (Wild type)	Worldwide	None
A	Africa	Mild
A-	Africa<br>South America	Mild-moderate
Mediterranean	Middle East, Europe, South Asia	Severe
Viangchan, Mahidol, Vanua Lava, Canton, Alant, Kaiping	Southeast Asia, Australasia	Mild-moderate-severe
Seattle	Mediterranean, Western Europe, North Africa	Mild-moderate
Union	Mediterranean, Western Europe, North Africa, China, Pacific Islands	Moderate-severe

5.3 Assessing a safe dose of PQ – preliminary data from Uganda

Since haemolysis is dose-dependent, side effects are expected to be less or absent when PQ is administered at concentrations below 0.75mg/kg. If a lower dose of PQ would be efficacious in clearing gametocytes, this would make a low dose of PQ a safe and powerful tool for community-wide malaria elimination strategies. Two studies in Thailand have suggested that lower doses of primaquine than the currently recommended 0.75 mg/kg can be equally efficacious in clearing gametocytes. Bunnag [9](1980) compared the effect of 15mg daily for 5 days, 30mg single dose and 45mg single dose in Thai adults and found no significant difference in gametocyte clearance between doses. Pukrittayakamee [10] compared 0.25mg/kg and 0.5mg/kg primaquine in adults and found both to have shorter gametocyte clearance times (GCT) than non-primaquine-containing regimens, with no significant difference in outcomes between the two doses of primaquine. A mass drug administration programme in Cambodia [11] used a 9mg stat dose of primaquine (approximately 0.15mg/kg) every ten days, with a significant reduction in microscopic gametocyte carriage from 13.1% to 0.8% after 3 years. These studies have shortcomings in their safety and

efficacy assessments. None of the studies included complete safety assessments and microscopy was used as gametocyte detection tool, which is notoriously insensitive for detecting low density gametocytes [12]. Furthermore, none of the studies determined the transmissibility of gametocytes persisting after PQ. These studies support the hypothesis that a lower, safer but efficacious dose of PQ may be found, but more detailed studies are clearly needed to provide conclusive evidence on the safety and efficacy of low dose PQ.

One of the studies that is providing this safety information is a study that is conducted in Jinja, Uganda by three of the researchers on this proposal. This trial is registered online at http://clinicaltrials.gov/ct2/show/NCT01365598 and will be completed in November 2012. In this study, that forms a sister trial for the current study, 480 children between 1-10 years are randomized to received artemether-lumefantrine alone or with 0.75, 0.4 or 0.1 mg/kg PQ. Unblinding will be done after completion of the trial but none of the 300 individuals who received either AL alone, AL+PQ (0.75mg/kg), AL+PQ (0.4mg/kg) or AL+PQ (0.1 mg/kg) as per 10/07/2012 showed signs of haemolysis.

In addition to safety assessments, some efficacy estimates are obtained. Gametocyte carriage after treatment is determined by molecular methods to determine the gametocyte clearance time. In Jinja, there are no insectary facilities that allow a direct determination of malaria transmission potential after ACT-PQ.

5.4 Mosquito feeding assays to confirm the efficacy of a lower dose of PQ

In general, there have been no studies that directly determined malaria transmission after ACT alone or in combination with PQ. A study from Tanzania found that very few gametocytes persisted several days after PQ and that the density of these low density gametocytes was probably too low to result in malaria transmission [2]. The viability of gametocytes can currently not be determined by microscopy or molecular tools. The infectiousness of gametocytes that are observed after treatment is highly variable, some drugs leaving highly infectious gametocytes unaffected [13], others inducing an efflux of immature and/or less infectious gametocytes [14, 15]. Moreover, low density gametocytes frequently result in mosquito infection [16].

Direct assessments of infectiousness are therefore needed to prove the efficacy of transmission blocking interventions. In the current study, we therefore propose to perform mosquito feeding assays to quantify transmissibility to mosquitoes at different time-points after PQ.

6. JUSTIFICATION OF THE STUDY

The current dose for a single dose of PQ of 0.75mg/kg dates back to the 1940s. There is sufficient evidence that this dose leads to haemolysis in G6PD deficient individuals who may comprise a significant proportion of the population in malaria endemic regions. A dose of PQ that is sufficiently high to effectively prevent onward malaria transmission but sufficiently low to be safe in all populations is urgently needed in the era of malaria elimination. In a sister project in Uganda that is currently finalising recruitment, low dose PQ was found to be safe in G6PD normal individuals. The current study will form the next step by assessing the lowest efficacious dose of PQ when given together with an ACT in a population with normal G6PD enzyme function who therefore have no increased risk of PQ-induced haemolysis. It will be the very first study to directly determine malaria transmission potential after ACT-PQ. The study will be conducted in asymptomatically infected individuals. Follow-up studies will confirm the efficacy of this dose in symptomatic patients and determine the safety profile in G6PD deficient individuals.

7. MAIN OBJECTIVE

To evaluate the efficacy and safety of different doses of primaquine administered with AL for the purpose of reducing *P. falciparum* gametocytes in the infected human host to prevent transmission of falciparum malaria to the anopheles mosquito vector.

8. SPECIFIC OBJECTIVES AND HYPOTHESES TO BE TESTED

1. To evaluate the efficacy of different doses of primaquine when administered with AL as measured by gametocyte prevalence and density
2. To evaluate the efficacy of different doses of primaquine when administered with AL as measured by mosquito feeding assays
3. To evaluate the safety of different doses of primaquine when administered with AL as measured by change in mean haemoglobin (Hb), prevalence of severe anaemia (Hb <5g/dL), and evidence of black urine (haemoglobinuria; dipstick positive)
4. To assess the safety of different doses of primaquine when administered with AL as measured by prevalence/ incidence of adverse events and tolerability

5. To obtain basic pharmacokinetic parameters for primaquine in the study population

9. DESIGN AND METHODOLOGY

9.1. General study design

The study is a randomized placebo-controlled trial with four parallel arms. A total of 480 individuals will be enrolled. Participants will be recruited from Saponé health district, near Ouagadougou. Screening lists are prepared based on census lists. If malaria parasites are detected at a density >1,000 parasites/µL in the absence of clinical symptoms suggesting malaria, individuals are eligible for enrolment. Prior to undergoing any study procedures, individuals will be screened by the study clinicians for eligibility to enter the study. If they satisfy initial criteria, individuals will be invited to give informed consent to participate in the clinical trial. Consenting participants will then undergo clinical and laboratory screening. If they satisfy the study selection criteria, they will be enrolled in the trial. A small minority may be excluded from the trial after day 0, when final laboratory screening results become available. If individuals do not satisfy selection criteria, their malaria infection will be managed by the local clinic staff.

All enrolled individuals will receive a full three-day course of AL, and will be randomized to receive a dose of primaquine or placebo with the fifth dose of AL on day 2. All doses of AL and PQ will be directly observed and randomization will commence with the comparator (AL-alone) arm and the lowest PQ dose. Sampling will be as follows: All individuals have finger prick blood samples on days 0, 2, 3, 7, 10 and 14 for malaria parasites (asexual and sexual), hb (using Hemocue®) and into an EDTA tube for gametocyte molecular analysis. On two occasions during this study period, either on day 3 and 10 or on day 7 and 14, , a venipuncture sample will be obtained for membrane feeding assays. On each day of follow up, there will be an assessment by a clinician and an assessment for adverse events. Participants will be reimbursed for travel to and from the clinic for all scheduled and non-scheduled visits during the time they are enrolled in the study. On day 1, every 4th child enrolled will be invited to consent for pharmacokinetic analysis on days 2-4. During follow up and beyond the last scheduled visit at day 14, all participants will be encouraged to attend the clinic for any medical concerns and the cost of travel to the clinic will be reimbursed.

9.2. Study site and population

The study will be carried out in the clinical trial field site of Balonghin in the health district of Saponé 45 kilometres Southwest of Ouagadougou. This site is being used for malaria vaccine trials and has an excellent infrastructure for clinical trials, including a well equipped clinical laboratory and wards dedicated to clinical trials.

Inclusion criteria:

1. Age > 2 years and <15 years
2. Weight over 10kg
3. *P. falciparum* parasitaemia >1,000 parasites and <200 000 parasites/µl
4. Normal G6PD enzyme function
5. Informed consent by legally acceptable representative

Exclusion criteria:

1. Enrolled in another study
2. Fever >37.5 degrees C (tympanic) or history of fever in the last 24 hours
3. Evidence of severe illness/ danger signs
4. Known allergy to study medications
5. Hb < 8g/dL
6. Started menstruation
7. Pregnancy or breastfeeding
8. Antimalarials taken within the last 2 days
9. Primaquine taken within the last 4 weeks
10. Blood transfusion within the last 90 days
11. Non-falciparum malaria co-infection

9.3 Participant recruitment and screening

Study subjects will be recruited from villages surrounding the Balonghin clinical trial centre. Census lists will be prepared to screen individuals in the proposed age-range who will have a blood slide taken. Screening blood smear slides will be read and counted by the outpatient laboratory technicians. Any participant with a positive screening thick smear will be referred to our clinic for further evaluation.

Upon referral to the study clinic, a standardized screening interview will be conducted by study physicians. This interview will go through the initial screening selection criteria that are given above. All individuals with fever or other symptoms suggestive of symptomatic malaria are excluded from study enrolment and referred back to the standard outpatient clinic for treatment of their malaria infection and other appropriate care.

9.4. Enrolment

Individuals who agree to participate for screening and meet all inclusion criteria will be invited for enrolment. During enrolment procedures, they will again be informed about the objectives and practical consequences of participation and asked to sign an informed consent form. A parent or guardian will be asked for consent. Children above 12 years of age will also be asked to provide assent. The possibility of withdrawal from the study, at any time and without any declaration of the reason will again be pointed out. They will then be invited to sign the written consent form adjoining the written study information and approved by the IRBs for their child to participate in a research study and a second consent for the future use of biological specimens obtained during the course of the study (Appendix A). If the parent or guardian is unable to read or write, their fingerprint will be used in substitute for a signature, and a signature from an impartial witness to the informed consent discussion will be obtained. Two copies of the consent form must be signed. The parent/ guardian/ impartial witness will sign/ fingerprint one copy for the study staff and one copy to keep for themselves. The assent form will be read through word for word and a witness signature will be requested.

Following the informed consent discussion, parents (or guardians) will be given their copy of the form to keep which includes the study information, the signed consent form and contact names and telephone numbers to use if they have further questions regarding the study or follow up procedures. If assent is obtained, the participant will keep their signed copy.

9.5. Randomization and blinding

After enrolment, participants will be assigned to a treatment group using a randomized method stratified by sex. The responsible study staff will select sequential opaque envelopes (from either the male or female pile). Each envelope contains a pre-determined treatment assignment code.

- The nurse selects next opaque envelope according to the child's gender. The envelope contains the participant's allocation code. The allocation code corresponds to one of the four treatment arms
- Study nurse labels envelope with the participant's study number and weight
- Study nurse presents the envelope to the pharmacist to request treatment. The pharmacist opens the envelope and documents the participant's study number and allocation code on the treatment assignment log
- Study pharmacist uses participant's weight and assignment code to calculate the correct primaquine dose or the equivalent dose of placebo to be given on day 2 (Appendix C)
- Study pharmacist logs this dose in the treatment assignment log together with the participant's treatment allocation code and study number. There is one treatment assignment log for each treatment arm. The study pharmacist calculates and documents the number of millilitres of PQ/ placebo solution that will need to be given on day 2
- The study pharmacist dispenses the six AL doses when the nurses request (morning and evening of days 0-2
- The study pharmacist labels the AL treatment assignment log form with the participant study number, treatment assignment code and AL batch number
- For all treatments (PQ/ placebo and AL), the study nurse will document that the treatment has been given, the number of tablets/ millilitres of drug given and whether or not the dose was vomited or repeated

9.6. Treatment and administration procedures

Children (n=480) will be randomized in a ratio 1:1:1:1 to treatment with:

i) <u>Artemether-lumefantrine alone:</u> artemether-lumefantrine (AL; Coartem®; Novartis Pharma) administered as administered as half a tablet (20 mg of artemether and 120 mg of lumefantrine) per 5 kg of body weight in a 6-dose regimen (at enrolment and 8, 20, 32, 44 and 56 h [±90 min] after initiation of treatment). The fifth dose is given together with a placebo that resembles the PQ solution.

ii) <u>AL-PQ1:</u> AL as described under i) administered as administered as half a tablet (20 mg of artemether and 120 mg of lumefantrine) per 5 kg of body weight in a 6-dose regimen (at enrolment and 8, 20, 32, 44 and 56 h [±90 min] after initiation of treatment) with PQ at 0.10mg/kg given together with the fifth dose of AL.

iii) <u>AL-PQ2:</u> AL as described under i) administered as administered as half a tablet (20 mg of artemether and 120 mg of lumefantrine) per 5 kg of body weight in a 6-dose regimen (at

enrolment and 8, 20, 32, 44 and 56 h [±90 min] after initiation of treatment) with PQ at 0.40mg/kg given together with the fifth dose of AL.

iv) AL-PQ3: AL as described under i) administered as administered as half a tablet (20 mg of artemether and 120 mg of lumefantrine) per 5 kg of body weight in a 6-dose regimen (at enrolment and 8, 20, 32, 44 and 56 h [±90 min] after initiation of treatment) with PQ at 0.75mg/kg given together with the fifth dose of AL.

For safety reasons, the first 80 participants will be randomized to arms i and ii in a ratio 1:1; participants 81-160 will be randomized to arms i, ii and iii in a ratio 1:1:2 and participants 161-480 are randomized to arms i, ii, iiii and iv in a ratio 3:3:4:6. This will give a final ratio of 1:1:1:1 but will allow assessment of the safety of the lower doses of PQ before a higher dose is administered.
All treatment is administered under supervision with fatty food to ensure adequate absorption and minimize the risk of gastro-intestinal side effects. All doses are given at the clinic under supervision of the study nurse.. For participants with anaemia (Hb < 10 gm/dL), we will follow Integrated Management of Childhood Illness (IMCI) and Burkina Faso national guidelines: anaemic children will be treated with iron sulfate (100 mg daily for 2 weeks) and mebendazole (250 mg age 1-2 years; 500 mg > 2 years age; treated no more frequently than every 6 months).

9.5. Follow-up and samples taken

Participants are asked to return to the clinic on various days: days 0 (initiation of treatment), 1, 2, 3 and 7, 10 and 14. On days 0, 1 and 2, participants visit the clinic for clinical examination and drug administration. On each day participants will be examined clinically and a structured questionnaire is used to determine the occurrence of side-effects of treatment (see appendix D). During screening and on day 2, 3, 7, 10 and 14 after initiation of treatment, participants will have a slide and Hb sample taken to determine whether asexual parasites and gametocytes have been cleared and if haemolysis occurs. A longer follow-up is not needed since there are no indications that PQ influences the clinical efficacy of AL and the study is purely design to determine safety and efficacy in the first two weeks after initiation of treatment. Hb concentration is determined by HemoCue photometer [Angelholm, Sweden]. Haemolysis is not expected based because G6PD deficient individuals are not enrolled but there are some indications that G6PD normal individuals may not be completely protected from PQ-induced haemolysis [5]. An RNA sample for gametocyte detection by quantitative nucleic acid sequence based amplification (QT-NASBA) will be collected on days 0, 2, 3,

7, 10 and 14 [2]. Biochemistry (ASAT, ALAT, total Bilurubin, Creatinin) and a full blood count are done for all individuals on days 0 and 7.

Each individual will be asked to participate in membrane feeding assays at two time-points in the fourteen day period: either days 3 and 10 or days 7 and 14. At each of these two occasions a venous blood sample of 3mL will be taken for membrane feeding assays, to collect nucleic acids for gametocyte detection by QT-NASBA and for the preparation of a blood slide.

9.6 Parasite detection

Blood smears are stained with 10% Giemsa for 10 minutes and then screened for asexual parasites and gametocytes at screening and on days 2, 3, 7 and 14 after initiation of treatment. Slides are double-read and read separately for asexual parasites and gametocytes in 100 microscopic fields. Parasite detection by Pfs25 QT-NASBA will be done using a NucliSens EasyQ analyser (bio-Mérieux) as described elsewhere for Pfs25 mRNA [16, 17]. Nucleic acids are extracted by automated extraction [Roche MagNapure] using commercial kits from 50µL blood samples. The *Pfs25* QT-NASBA technique is gametocyte specific and has an approximate detection limit of 0.1 gametocytes/µL in the blood volume taken. NucliSens Basic kits are used for amplification in accordance with the manufacturer's instructions. A standard dilution series of mature, *in vitro* cultured NF54 gametocytes [17] is included in each run. Gametocyte detection by *Pfs25* QT-NASBA is done for all samples on days 0, 2, 3, 7, 10 and 14 after initiation of treatment.

Table 1. Sampling schedule

**Day of follow up**	**0**	**1**	**2**	**3**	**7**	**10**	**14**	**Unscheduled**
**<u>CLINICAL:</u>**								
History	**X**	**X**	**X**	**X**	**X**	**X**	**X**	**X**
Tympanic temperature	**X**	**X**	**X**	**X**	**X**	**X**	**X**	**X**
Physical examination	**X**	**X**	**X**	**X**	**X**	**X**	**X**	**X**
Assessment for adverse events	**X**	**X**	**X**	**X**	**X**	**X**	**X**	**X**
Complete case record form	**X**	**X**	**X**	**X**	**X**	**X**	**X**	**X**
**<u>TREATMENT:</u>**								
AL	**X (1,2)**	**X (3,4)**	**X (5,6)**					
PQ			**X (with 5)**					

LAB TESTING:								
Blood smear	X		X	X	X	X	X	X
Filter paper W#3 + W#903	X		X	X	X	X	X	X
RNA sample	X		X	X	X	X	X	
Membrane feeding (2 of these 4 days)				X	X	X	X	
Haemoglobin (Hemocue®)	X		X	X	X	X	X	
G6PD function	X							

9.7 Pharmacological analysis

Pharmacokinetic evaluations will be obtained on approximately one quarter of the enrolled participants; a maximum of 160 participants will be recruited for pharmacokinetic sampling. There will be a separate consent process for this evaluation. Participants will be consented for this on day 1 and asked to come for sampling on days 2 to 4. The sampling on day 2 will happen whilst they are at the clinic for their last day of AL and the study dose of PQ/ placebo.

The pharmacokinetic sampling will involve taking a total of 7 venous blood samples of less than 2mls. The total amount sampled, being approximately 11-14 mls in 3 days. The first sample is just prior to the PQ/ placebo dose (a baseline sample) and the subsequent six doses are at intervals up to 72 hours after the dose of primaquine/ placebo. The blood samples will be taken at fixed times between 8am to 5pm. Participants will have to attend the clinic a minimum of 30 minutes prior to this to enable preparation for sampling. The first 5 samples are taken on day 2 and they will be taken through a venflon, sited when the baseline pharmacokinetic sample is taken. If a venflon is not sited successfully, a butterfly needle may be used. The last two samples (one on day 3 and one on day 4) will be taken by individual blood draws (venipuncture). The participant will be asked to stay in the clinic between sampling times on day 2.

In order to minimize the total number of blood draws per participant, the sampling timeframe has been randomized so that over the total population of participants, a population pharmacokinetic model can be constructed for analysis. Six randomized sample times will be allocated to sequential consenting participants in opaque envelopes. Each sample time is within a window so that there are 5 samples on day 2 and one each on days 3 and 4. Pharmacokinetic samples will be analysed in Professor Niklas Lindegardh's laboratory in Mahidol University, Bangkok, Thailand, where the randomized sampling framework was generated.

Selection for pharmacokinetic analysis

On day 1, every fourth child who was enrolled will be invited to give written informed consent for pharmacokinetic sampling (Appendix E). As far as possible, children will be seen in enrolment order (study number order) on each day. If this child declines consent, the next consecutive participant will be invited. The consent interview will be conducted by study clinicians. The pharmacokinetics consent form will be attached to an information leaflet in the appropriate language and it will be read word for word to the guardian of the child. Children over the age of twelve years will be invited to give written assent by signing a form with attached information sheet which is read to them.
All participants undergoing pharmacokinetic sampling must satisfy the following criteria:

Inclusion criteria:

1. Haemoglobin >8g/dl
2. Siting of secure blood sampling access feasible (venflon/ butterfly needle) on day 2
3. Willing and able to attend study clinic by 7.30am on days 2-4
4. Willing to stay on study clinic premises between 8am to 5pm on day 2

Consenting participants will be seen by a study clinician who will select the next available pharmacokinetic opaque envelope. This will contain a sheet with the sampling times for the participant. The clinician will label this sheet with the participant's study number. The clinician will be responsible for adhering to the sampling times.
The clinician will fix venous access (using a venflon or butterfly) and take the baseline sample. All blood will be taken into heparinised tubes. The subsequent 4 samples will be taken at the allocated sampling times (Table 2). Then the fixed venous access will be removed. The participant will be informed as to what time they need to present by on days 3 and 4 for the last two samples. On these days, the sample will be taken by phlebotomy with no fixed access.

Table 2 Sampling framework for participants recruited to pharmacokinetic studies

**Day of follow up**	0	1	2	3	4	7	10	14	unscheduled
PQ pharmacokinetic sampling windows:*<br><br>(baseline plus x 6 windows)<br><br>Serum samples			0<br><br>0-2<br><br>2-3<br><br>3-6<br><br>6-9	24-33	48-72				

*each participant has one sample within each sampling window

9.8 Haematology and biochemistry

During screening, haemoglobin will be assessed with a finger prick blood sample using a HemoCue® photometer (Ängelholm, Sweden). This produces a point-of-care result. If clinically indicated during follow-up, a venepuncture sample will be taken for a full blood count (haemoglobin, white blood cell count, platelets and haematocrit) and biochemistry. On days 0 and 7, and on any day when the clinician feels this is required for the investigation of adverse events, biochemistry samples will be taken. These samples will be used for a full blood count and biochemistry including, for example, renal and liver function testing and urinalysis for haemolysis. These samples will be sent to a commercial laboratory with quality control systems.

9.9 G6PD deficiency testing

An EDTA tube or heparinized haematocrit capillary tube will be used to acquire blood for glucose-6-phosphate-dehydrogenase semi-quantitative fluorescent spot test for initial screening in the clinic site. This will require approximately 0.5 ml of blood. A reagent solution containing Glucose-6-P + NADP+ is mixed with whole blood or a dried blood spot. Samples obtained from normal or slightly reduced G6PD activity will show strong fluoresce. Failure to fluoresce after 10-minutes of incubation suggests a total or marked deficiency of G6PD. This test may fluoresce falsely if the study participant has had a blood transfusion within the last 90 days hence, these persons will be excluded from the study. In addition, dried blood spots obtained on filter paper will be stored for quantitative G6PD testing. This will be performed using the G-6-PD OSMMR 2000 kit [R&D Diagnostics]. The dried blood spots will also be utilized for G6PD genotyping.

9.10 Membrane feeding assays

Participants will be invited for membrane feeding assays on different time-points. Individuals who participate in pharmacological analyses are excluded from this procedure to minimize the total volume of blood taken per individual. All other individuals will be invited to participate in membrane feeding assays twice (two of the days 3, 7, 10 and 14). Membrane feeds are done for all individuals who are willing to participate in this part of the study and sign an additional informed consent section (Appendix A). A maximum of 8 experiments is done per day for mosquito husbandry reasons. For each experiment, a 3mL venous blood sample is obtained and fed to 100 locally reared 4 to 5-day-old female *An. gambiae s.s.* mosquitoes via an artificial membrane attached to a water-jacketed glass feeder maintained at 37°C. After exactly 15 minutes, fully and partially fed mosquitoes are

selected and kept on glucose for 10 days at 27°C–29°C. Mosquitoes that die during this 10 day period are stored on silicagel for detection of oocysts by circumsporozoite ELISA. Mosquitoes that survive until day 10 are killed and stored on silicagel for detection of oocysts by circumsporozoite ELISA. It is considered unethical to keep mosquitoes in the insectary beyond 10 days after feeding on a parasitaemic blood meal since mosquitoes may develop sporozoites and become infectious to humans after this time-point, creating a health hazard for laboratory workers.

10. SAMPLE SIZE CONSIDERATIONS

The number of participants required in each treatment arm was calculated for each of the two primary outcome measures:

1. EFFICACY PRIMARY OUTCOME MEASURE: number of days to gametocyte clearance (gametocyte clearance time, GCT)
2. SAFETY PRIMARY OUTCOME MEASURE: maximal fall (+/ or -) in haemoglobin (g/dL) from enrolment to day 14 of follow-up

10.1 Sample size for efficacy

For efficacy, the sample size calculation is based on non-inferiority of each of the two test dose arms to the comparator arm, the WHO-recommended dose of PQ, 0.75mg/kg.

The non-inferiority margin for days to gametocyte clearance is proposed as 2.5 days, taking into consideration data from previous studies. The addition of primaquine to ACT in Tanzania reduced the time to gametocyte clearance from 28.6 to 6.3 days. We used the size of this difference to consider a clinically acceptable inferiority margin. The standard deviation for time to gametocyte clearance is estimated as 6 days [4]. Applying these assumptions, and allowing for a 10% loss to follow up, a sample size of 120 per arm will provide over 80% power at the 0.05 significance level to detect non-inferiority to the standard arm with a non-inferiority margin of 2.5.

This sample size also allows for an analysis of superiority of the efficacy of the two test dose arms to placebo.

10.2 Sample size for safety

For safety, the sample size calculation is based on superiority of each of the two test dose arms to the comparator arm, the WHO-recommended dose of PQ, 0.75mg/kg. The overall mean absolute drop in Hb by day 7 after treatment with AL-PQ is expected to be 0.6g/dL with a standard deviation of 1.5 [5]. Therefore, with 80% power and at the 0.05 significance level, a sample size of 99 would be required to detect a difference in mean maximal drop in Hb between treatment groups of 0.6g/dL.

Therefore, a total study size of 480 will be required to analyse both of the primary outcomes.

11. DATA ANALYSIS PLAN

For each treatment group, the numbers of participants who were randomised, received each dose of the intended treatment, and were analysed for the primary outcome will be represented in a CONSORT flow chart. The mean and standard deviation of the number of days to gametocyte clearance (gametocyte clearance time; GCT) will be estimated in each treatment arm by use of a mathematical model [4].

For each of the two test low PQ dose treatment arms, a 95% confidence interval for the difference in mean GCT between the test arm and the WHO-recommended PQ dose treatment arm (0.75mg/kg) will be calculated (mean GCT in test arm – mean GCT in PQ 0.75mg/kg arm). For each of the two low dose PQ arms, superiority over the AL alone arm will be assessed using unpaired t-tests. Differences between means and 95% confidence intervals for the differences will be calculated. The proportion of infected mosquitoes and oocyst burden in infected mosquitoes will be compared between treatment arms, adjusting for observations derived from the same individual.

The primary safety outcome, maximal fall (+/ or -) in haemoglobin (g/dL) compared to enrolment value during follow-up, is expressed as an arithmetic mean (+/- standard deviation) per treatment arm and pair-wise comparisons made between each of the two test low PQ dose treatment arms and the comparator (0.75 mg/kg WHO-recommended) arm using unpaired t-tests. Differences between means and 95% confidence intervals will be calculated.
Population pharmacokinetic modelling analysis is under design by collaborators in Mahidol Oxford Research Unit, Bangkok, Thailand. The sampling framework has been optimised to minimise sampling points, to prevent the need for overnight stay at the clinic and to produce basic pharmacokinetic parameters including: AUC of concentration over time, T_{max}, C_{max}, oral clearance, terminal half life of primaquine +/- metabolites.

12. ETHICAL CONSIDERATIONS

This study specifically excludes G6PD deficient individuals who are at an increased risk of haematolytic side-effects of PQ. Within this population, safety of the AL-PQ combination will be

extensively studied. This information is essential to justify further exploration of this drug combination for malaria control and elimination in populations.

The current study will restrict enrolment to asymptomatically infected individuals. In follow-up studies symptomatic malaria cases will be enrolled.

Blood sampling will be restricted to the absolute minimum to answer the study questions. Both pharmacological and membrane feeding sampling schedules are chosen to minimize the number of sampling time-points for individual participants while ensuring complete coverage of relevant time-points within a population (e.g. membrane feeding experiments are conducted for each individual on two of the time-points 3, 7, 10 and 14). Moreover, children will not participate in both pharmacological and membrane feeding assessments to minimize the total volume of blood taken. The total blood volume that is taken per participant during their 14-day period of participation will be ~14mL for children selected for pharmacological analyses (25% of the study population) and ~7mL for individuals who are not included in the pharmacological analyses but are willing to participate in membrane feeding assays.

12.1 Confidentiality

All participant records are to be used only for the purpose of this research project. Names will not appear on labels on laboratory specimens or in any report resulting from the study.

Identifying information will be kept in a metal cabinet that is locked and only accessible to the study clinician or his clinical representative in case he is absent. Paper forms are only accessible to senior research staff (i.e. clinician, nurse and scientific personnel but not field workers or laboratory assistants); names and addresses will be removed from digital files which will be password protected. All materials collected in this study will be labelled with a study identification number that cannot be directly linked to identifying information.

12.2 Participant reimbursement

Participants will receive a small parcel with items such as sugar, rice and flour on a daily basis to compensate them for the time lost during the study. This is common practice for all clinical trials at CNRFP.

12.3 Good clinical practice

The study will be conducted in accordance with the latest South Africa revision of the Declaration of Helsinki and local regulatory requirements, and study staff will receive a local workshop in Good Clinical Practice before the study starts. AL is the first line antimalarial drug in Burkina Faso; PQ is a registered drug that has been extensively used over the last 50 years and is recommended by the

WHO. A data safety and monitoring board will be installed to ensure the trial is completed according to GCP standards.

13. SAFETY MANAGEMENT

13.1 Safety management

13.1.1 Withdrawal of individual subjects

Subjects can leave the study at any time for any reason if they wish to do so without any consequences. The principal investigators and trial clinicians can decide to withdraw a subject from the study for urgent medical reasons.

Subjects can be withdrawn from the study procedures for the following reasons:

- Any serious adverse event (AE)
- Any AE that, according to clinical judgment of the trial clinicians, is considered as a definite contra-indication to proceeding with the study procedures
- Withdrawal of informed consent by subject
- Failure to comply with the prescribed study protocol or complete loss to follow-up
- Repeated vomiting within 30 minutes after administration of study drug

13.1.2 Premature termination of the study

The study may be discontinued for the following reasons:

- On advice of the local safety monitor
- On advice of the data safety monitoring board (DSMB)
- On advice of the principal investigators and trial clinicians
- On advice of the ethics committee

The local safety monitor, DSMB, principal investigators, trial clinicians or ethics committee may decide to put the study on hold based on adverse events, pending discussion with the local safety monitor / DSMB / principal investigators / trial clinicians / ethics committee. Following discussion, it may be decided to terminate the study.

13.2 Management of haemolysis

In previous studies where a single dose of 0.75mg/kg primaquine has been administered, the incidence of severe haemolysis has been low. In Tanzania [2], none of the participants experienced symptoms of anaemia and no child required a blood transfusion. In the second study in Tanzania [5], one G6PD deficient child who received primaquine 0.75mg/kg had severe anaemia, but did not require a blood transfusion and recovered with haematinic drug treatment.

In Sudan [18], there were no severe or serious or adverse events and severe anaemia was not reported.

Consequently, given that the frequency of G6PD deficiency is likely to be similar in Uganda, and children with low G6PD enzyme function on day 0 are excluded from enrolment we do not expect haemolysis to occur frequently in those participants receiving 0.75mg/kg of primaquine. We predict that those participants in the dose arms lower than 0.75mg/kg should have an even lower chance of developing haemolysis because haemolysis is dose-related.

For the purposes of systematic and responsible safety monitoring, the following detailed protocols have been developed for the management of participants in whom haemolysis is suspected.

13.2.1 Measures of haemolysis

Haemolysis will be suspected according to criteria in a study SOP, detailing the size of haemoglobin fall (measured by Hemocue®) after PQ/ placebo treatment and the absolute haemoglobin value. In addition, any child presenting with or complaining of dark or black urine will be assessed for haemolysis.If haemolysis is suspected, a venepuncture sample will be taken for a full blood count and G6PD enzyme function, a blood film will be prepared and analysed for schistocytes, urine dipstix will be taken and a clinical examination performed.

Anaemia

Participants with a haemoglobin below 10g/dL (according to IMCI guidelines) will be treated with haematinic drugs and de-worming according to IMCI and national guidelines.

Haemolysis

Children with evidence of mild haemolysis will be observed and monitored and haematinic drugs will be considered according to national guidelines. Haemoglobin testing will be repeated according to the child's clinical progress. Any child reporting black urine will be assessed by a study physician. Black urine is defined as any dark-coloured urine with brown or black pigments (not orange). A full clinical exam and history will be obtained, a urine sample sent for analysis (haematuria, protein) and a venous blood sample drawn for assessment of full blood count (FBC) and renal function (including bicarbonate). A blood film will be assessed for schistocytes. Requirement for blood transfusion will be assessed by physicians, considering the size and rate of Hb drop and signs of clinical compromise, according to national guidelines. If blood transfusion is required, the participant will be transferred to a district Hospital of Saponé.

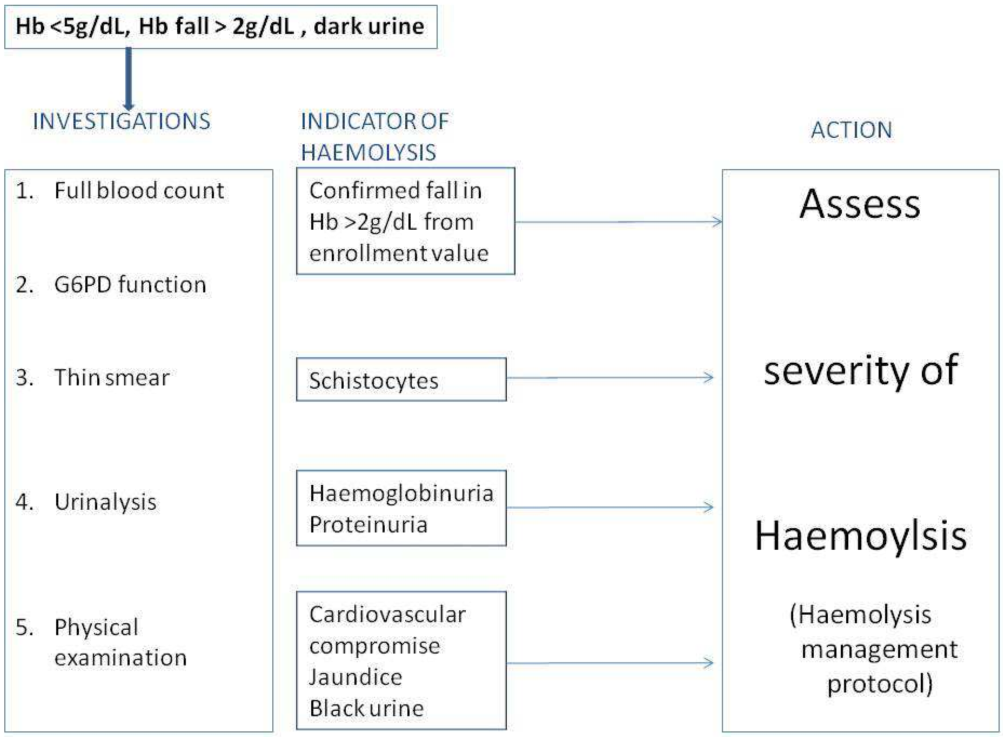

Figure 1. Investigation of haemolysis

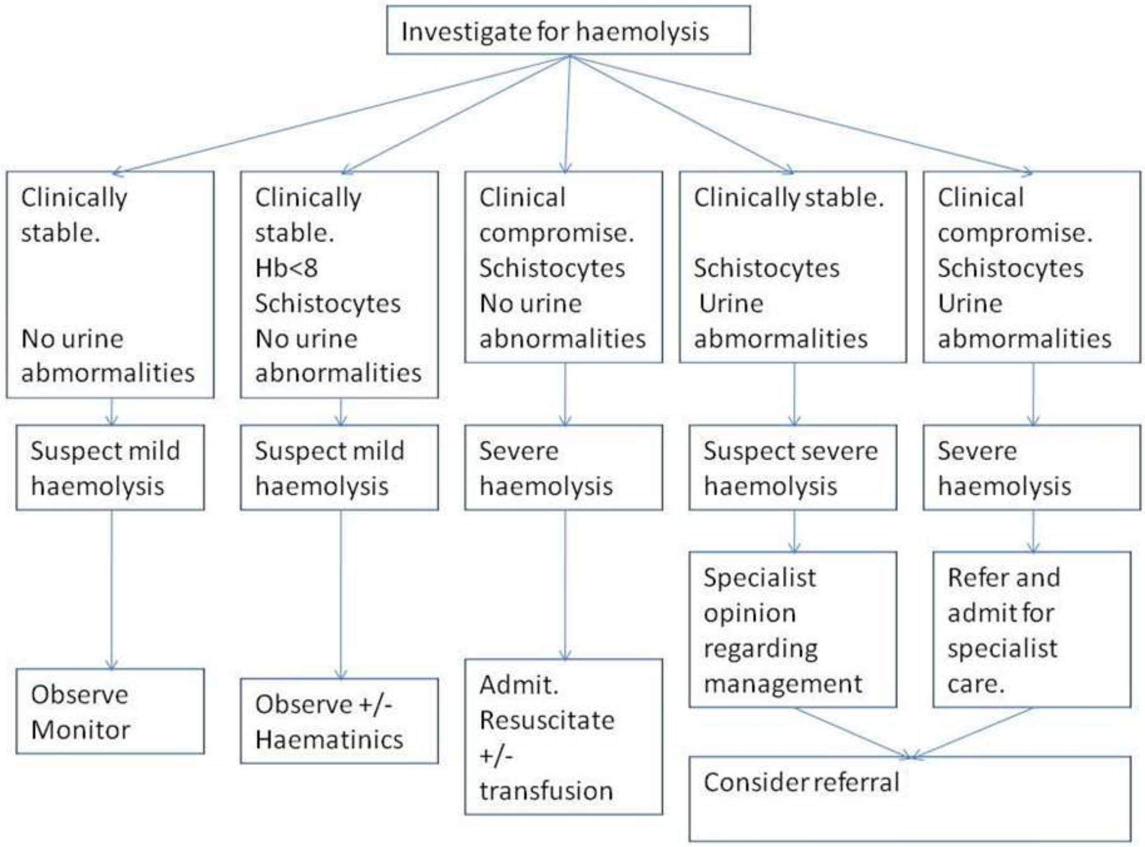

Figure2. management of haemolysis

13.3 Safety reporting

13.3.1 Adverse events

Adverse events are defined as any undesirable experience occurring to a subject during the study, whether or not considered related to trial. An AE can therefore be any unfavourable and unintended sign (including an abnormal laboratory finding), symptom, or disease (new or exacerbated) temporally associated with a study intervention. AEs may include events that occur as a result of protocol-mandated procedures (i.e. invasive procedures). All AEs reported spontaneously by the subject or observed by the trial clinicians or their staff will be recorded.

Abnormal laboratory findings or other abnormal assessments that are judged by the trial clinicians to be clinically significant will be recorded as AEs or serious adverse events (SAEs) if they meet the definition. The principal investigators and trial clinicians will exercise their medical and scientific judgement in deciding whether an abnormal laboratory finding or other abnormal assessment is clinically significant.

13.3.2 Serious adverse events

A serious adverse event is any untoward medical occurrence or effect that at any dose:

- Results in death
- Is life threatening (at the time of the event)
- Requires hospitalisation
- Results in persistent or significant disability or incapacity
- Is a new event of the trial likely to affect the safety of the subjects, such as an unexpected outcome of an adverse reaction or a major safety finding from a newly completed animal study etc.Additionally, Haemoglobin values drop of ≥ 40% of baseline haemoglobin and /or all blood transfusions, and /or all haemoglobin values of <= 5g/dL, if they occur will be reported as SAEs

All SAEs will be reported immediately to the principal investigators. The latter will subsequently report to the Sponsor (London School of Hygiene and Tropical Medicine) within 24hrs of awareness and to the chairpersons of the DSMB and Ethics Committee. (for more information: sections 13.3.3 and 13.4)

13.3.3 Adverse Event Data Collection

Safety assessments will be performed, and recorded by the trial clinicians. All AEs/reactions, observed by the trial clinicians or by the subject, will be accurately documented in the case report form. For each event/reaction the following details will be recorded:

1. Description of the event(s)/reaction(s)
2. Date and time of occurrence
3. Duration
4. Intensity
5. Relationship with the intervention
6. Action taken, including treatment
7. Outcome

In addition, symptoms will be ranked as (1) mild, (2) moderate, or (3) severe, depending on their intensity. All AEs except fever will be judged for their intensity according to the following scale:

- Mild (grade 1): awareness of symptoms that are easily tolerated and do not interfere with usual daily activity
- Moderate (grade 2): discomfort that interferes with or limits usual daily activity
- Severe (grade 3): disabling, with subsequent inability to perform usual daily activity, resulting in absence or required bed rest

For fever, the following scale will be used:

- Mild (grade 1): 37.5 - 38.0°C
- Moderate (grade 2): > 38.0 to 39.0°C
- Severe (grade 3): > 39.0°C

If an AE changes in frequency or intensity during the specified reporting period, the previous description of the AE will be corrected.

When an AE/SAE occurs, it is the responsibility of the principal investigators and trial clinicians to review all documentation (e.g. hospital progress notes, laboratory, and diagnostics reports) related to the event. The principal investigators and trial clinicians will then record all relevant information regarding an AE/SAE on the CRF or SAE Report Form, respectively. Furthermore, the trial clinicians

will attempt to establish a diagnosis of the event based on signs, symptoms, and/or other clinical information. In such cases, the diagnosis should be documented as the AE/SAE and not the individual signs/symptoms.

13.3.4 Assessment of causality

The principal investigators and trial clinicians are obligated to assess the relationship between study procedures and the occurrence of each AE/SAE. The clinicians will use clinical judgment to determine the relationship. Alternative causes, such as natural history of the underlying diseases, concomitant therapy, other risk factors and the temporal relationship of the event to the study procedures will be considered and investigated. The relationship of the AE with the study procedures will be categorized as:

Definite	An AE that follows a clear cut temporal association with a positive re-challenge test of laboratory confirmation
Probable	An AE that follows a reasonable temporal sequence from the study procedures and cannot be reasonably explained by the known characteristics of the subject's clinical state.
Possible	An AE for which insufficient information exists to indicate a high improbability that the event is related to the study procedures.
Not related	An event for which sufficient information exists to indicate that the etiology is unrelated either because of the temporal sequence of events or because of the subject's clinical state or other therapies.

13.4 Safety follow-up

13.4.1 Follow-up of subjects that voluntarily withdraw participation

If a subject fails to appear for follow-up, effort will be undertaken to locate or recall him/her or at least to determine his/her health status. These efforts will be documented in the subject's CRF and source documents.

In the event that a subject discontinues the study for any reason, the trial clinicians will conclude appropriate safety assessments for the subject and, in the case of an (S)AE, which has not yet resolved, they will schedule follow-up visits until the AE resolves or stabilizes.

13.4.2 Follow-up of adverse events

All AEs will be followed until they have abated, or until a stable situation has been reached. Depending on the event, follow-up may require additional tests or medical procedures as indicated, and/or referral to other specialists as the trial clinicians see fit.

The trial clinicians will follow-up subjects:

- with SAEs or those withdrawn from the study as a result of an AE, until the event has resolved, subsided, stabilized, disappeared, the event is otherwise explained, or the subject is lost to follow-up.
- with other non-serious AEs, until the subject has completed the study or is lost to follow-up.

Clinically significant laboratory abnormalities will be followed up until they have normalized, or until an alternative explanation, that is not related to the study has been provided. Additional information (including but not limited to laboratory results) relative to the subsequent course of such an abnormality will be made available to the local safety monitor.

13.4.3 Follow-up of serious adverse events

In the case of a suspected SAE, the nurses or trial clinicians will immediately report this occurrence to the principal investigators (PIs). The latter will subsequently report to the Sponsor (London School of Hygiene and Tropical Medicine) within 24hrs of awareness and to the chairpersons of the DSMB and Ethics Committee. In their report, the PIs will provide as much details as possible about the suspected SAE. At the same time the PIs, in consultation with the trial clinicians will stop any administration of study drugs, if the SAE is thought to be as a result of the study drug. The trial clinicians and consultant clinicians will immediately institute appropriate medical treatment to the subject who develops an SAE until the event has resolved, subsided, stabilized, disappeared, or the event is otherwise explained.

All findings of investigations and treatments administered will be recorded on the subject's CRF and also on the SAE Report Form. The trial clinicians may also refer the subject to other specialists as they see fit. The PIs will forward the completed SAE form to the local safety monitor to assess whether the SAE could have been a result of the study procedures, as per standard criteria. Findings of this assessment will be forwarded to the sponsor (LSHTM) and to the chairpersons of the DSMB and Ethics Committee.

SAE reported during the trial will be assessed and classified as:

1. Recovered/resolved

2. Not recovered/not resolved
3. Recovering/resolving
4. Recovered with sequelae/resolved with sequelae
5. Fatal (SAEs only)

13.5 Local Safety Monitor and Data Safety Monitoring Board (DSMB)

For this study, a local safety monitor is appointed, who is based in the Saponé and will be involved in the review of SAEs and subject safety. He is an experienced clinician and independent of the investigators team. His main responsibility will be the assessment of the events and recommendation regarding halting further study procedures.

Furthermore, an independent DSMB has been appointed, which consists of four skilled professionals constituted according to sponsor procedures that respect international standards. These experts will monitor the progress of the study with particular interest in the safety of trial subjects.
The DSMB will be responsible for:

- Reviewing safety and follow-up in an unblinded manner
- Reviewing reports on SAEs when they occur
- Making recommendations to the sponsor on the safety balance between comparison groups
- Making recommendations regarding continuing, amending or termination of the study for safety reasons

The local safety monitor and DSMB will receive progress reports on a weekly basis by email. These reports will be prepared by the PIs and trial clinicians and include a list of all reported AEs and any safety laboratory values (e.g. Hb, ALAT/ASAT) outside the normal range.

All SAEs will be reported by the PIs to the Sponsor (LSHTM), the local safety monitor, the chairpersons of the DSMB and Ethics Committee within 24 hours of awareness. The DSMB is empowered to suspend the enrolment to the trial pending review of potential safety issues.

13.6 Data monitoring

A local independent data monitor from CNRFP will undertake data monitoring at site (e.g. source data verification). In addition, there will be central monitoring by a monitor that will be appointed in consultation with the sponsor. This central monitoring will undertake statistical monitoring of inliers and outliers, data management review etc.

14. TIME FRAME AND DURATION OF THE PROJECT

Data collection will take place in 2012-2013. The enrolment is envisaged to take 4-6 weeks, the clinical follow-up another two weeks after enrolment is completed. Community sensitization is expected to start in October 2012, the data collection will take place between October 2012-January 2013. The project is followed by a community meeting to present some of the preliminary findings. Data analysis, laboratory analysis on mosquito and RNA samples will take place in the first 6 months of 2012.

15. REFERENCES

1. World Health Organization: Malaria elimination: a field manual for low and moderate endemic countries. 2007.
2. Shekalaghe S, Drakeley C, Gosling R et al. Primaquine Clears Submicroscopic Plasmodium falciparum Gametocytes that Persist after Treatment with Sulphadoxine-Pyrimethamine and Artesunate. PLoSONE 2007, 2:e1023.
3. Smithuis F, Kyaw MK, Phe O, et al. Effectiveness of five artemisinin combination regimens with or without primaquine in uncomplicated falciparum malaria: an open-label randomised trial. Lancet Infect Dis 2010; 10:673-681.
4. Bousema T, Okell L, Shekalaghe S, et al. Revisiting the circulation time of Plasmodium falciparum gametocytes: molecular detection methods to estimate the duration of gametocyte carriage and the effect of gametocytocidal drugs. Malar J 2010; 9:136.
5. Shekalaghe SA, ter Braak R, Daou M, et al. In Tanzania, hemolysis after a single dose of primaquine coadministered with an artemisinin is not restricted to glucose-6-phosphate dehydrogenase-deficient (G6PD A-) individuals. Antimicrob Agents Chemother 2010, 54:1762-1768.
6. Baird JK, Surjadjaja C. Consideration of ethics in primaquine therapy against malaria transmission. Trends Parasitol. 2011;27(1):11-6
7. Tiono AB, Dicko A, Ndububa DA et al. Chlorproguanil-dapsone-artesunate versus chlorproguanil-dapsone: a randomized, double-blind, phase III trial in African children, adolescents, and adults with uncomplicated Plasmodium falciparum malaria. Am J Trop Med Hyg. 2009;81(6):969-78.
8. Carter N, Pamba A, Duparc S, Waitumbi JN. Frequency of glucose-6-phosphate dehydrogenase deficiency in malaria patients from six African countries enrolled in two randomized anti-malarial clinical trials. Malar J. 2011;10:241.
9. Bunnag D, Harinasuta T, Pinichpongse S, Suntharasami P. Effect of primaquine on gametocytes of Plasmodium falciparum in Thailand. Lancet. 1980;2:91
10. Pukrittayakamee S, Chotivanich K, Chantra A et al. Activities of artesunate and primaquine against asexual- and sexual-stage parasites in falciparum malaria. Antimicrob Agents Chemother. 2004;48(4):1329-34.

11. Song J, Socheat D, Tan B, et al. Rapid and effective malaria control in Cambodia through mass administration of artemisinin-piperaquine. Malar J. 2010;9:57.
12. Bousema T, Drakeley C. Epidemiology and infectivity of Plasmodium falciparum and Plasmodium vivax gametocytes in relation to malaria control and elimination. Clin Microbiol Rev 2011; 24:377-410.
13. Fofana B, Djimde AA, Sagara I, et al. Impact of artemisinin-based combination therapy on malaria transmission in Mali. 5th MIM Pan-African Malaria Conference. Nairobi, Kenya, 2009:MIM16762172.
14. Targett G, Drakeley C, Jawara M, et al. Artesunate reduces but does not prevent posttreatment transmission of Plasmodium falciparum to Anopheles gambiae. Journal of Infectious Diseases 2001; 183:1254-1259.
15. Hallett RL, Dunyo S, Ord R, et al. Chloroquine/sulphadoxine-pyrimethamine for gambian children with malaria: transmission to mosquitoes of multidrug-resistant Plasmodium falciparum. PLoSClinTrials 2006; 1:e15.
16. Ouedraogo AL, Bousema T, Schneider P, et al. Substantial contribution of submicroscopical Plasmodium falciparum gametocyte carriage to the infectious reservoir in an area of seasonal transmission. PLoS One 2009; 4:e8410.
17. Schneider P, Schoone G, Schallig H, et al. Quantification of Plasmodium falciparum gametocytes in differential stages of development by quantitative nucleic acid sequence-based amplification. MolBiochemParasitol 2004; 137:35-41.
18. El-Sayed B, El-Zaki SE, Babiker H et al. A randomized open-label trial of artesunate-sulfadoxine-pyrimethamine with or without primaquine for elimination of sub-microscopic P. falciparum parasitaemia and gametocyte carriage in eastern Sudan. PLoS One. 2007;2: e1311.

16. ROLES INVESTIGATORS

Sodiomon Sirima, MD PhD is a clinician and senior researcher and the local PI of this project. He has coordinated several clinical trials in Burkina Faso, including multiple trials with antimalarial drugs and a recent malaria vaccine trial.

André Lin Ouédraogo PhD, is a post-doctoral scientist who will coordinate membrane feeding assays. He has coordinated several community studies on the human infectious reservoir in 2003-2011.

Guido Bastiaens, MD is a trial clinician. He was the trial physician for several studies involving experimental malaria infections in human volunteers at RUNMC. He will support data enrolment and clinical management.

Chi Eziefula, MD is a trial clinician who conducted the sister project in Uganda. She will support protocol development and clinical management.

Alfred Tiono, MD, PhD is an experienced trial clinician who coordinated several clinical trials at CNRFP including a clinical trial with AL-ivermectin to interrupt malaria transmission.

Teun Bousema, PhD is the international principal investigator. He is a senior epidemiologist at RUNMC and LSHTM and has coordinated clinical trials with antimalarial drugs and malaria transmission endpoints in Kenya (2003-2004) and Tanzania (2004-2008). He will coordinate data collection and analysis.

Chris Drakeley, PhD is a field epidemiologist with a long-standing track record on the assessment of transmission potential after antimalarial drug combinations.
