# Supplementary material for: Single low dose primaquine to reduce gametocyte carriage and Plasmodium falciparum transmission after artemether-lumefantrine in children with asymptomatic infection: a randomised, double-blind, placebo-controlled trial
Source: BMC Med. 2016 Mar 8;14:40. doi: 10.1186/s12916-016-0581-y (PMC4782330; doi:10.1186/s12916-016-0581-y)
Supplement: Additional file 3: — Supplementary Appendix. (PDF 102 kb) [file 12916_2016_581_MOESM3_ESM.pdf]

Supplementary Appendix

Single Low Dose Primaquine to Reduce Gametocyte Carriage and *Plasmodium falciparum* Transmission after Artemether-Lumefantrine in Children with Asymptomatic Infection: A Randomised, Double-Blind, Placebo-Controlled Trial

Authors: Gonçalves BP, Tiono AB, Ouédraogo A, Guelbéogo WM, Bradley J, Nebie I, Siaka D, Lanke K, Eziefula AC, Diarra A, Pett H, Bougouma EC, Sirima SB, Drakeley C, Bousema T

Non-linear model to estimate gametocyte clearance time

A non-linear model was fit in SAS 9.3 (SAS Institute, North Carolina, United States) to estimate the rate of gametocyte clearance in study participants allocated to different treatment regimens:

$$G_t = e^{-rt} \tag{1}$$

where G_t represents the proportion of individuals with gametocytes at time t. r is the rate with which gametocytes are cleared and depends on the treatment received:

$$r = e^{(\ln r_0 + aI_1 + bI_2)} \tag{2}$$

where r_0 corresponds to the clearance rate in the artemether-lumefantrine only group; and I_1 and I_2 are indicator variables representing the 0.25 mg/kg and the 0.40 mg/kg primaquine dose arms, respectively. a and b represent the effects of the different primaquine doses on the rate of gametocyte clearance and are estimated from the data.

Haematological changes after treatment

Finger-prick measurements

219 children had haemoglobin levels quantified by Hemocue photometer on day 0. For those study subjects without Hemocue-derived haemoglobin levels in the beginning of the follow-up, measurements done at screening were used as baseline: the median (interquartile range) interval between screening and the day 0 of follow-up was 2 (1 – 2) days. The majority (227/360) of study participants had haemoglobin levels measured on five follow-up visits after day 0; 9 children had 2 or less measurements. When all enrolled individuals are considered, regardless of the number of Hemocue measurements during follow-up, the average (95% CI) maximal fall in haemoglobin concentrations after treatment initiation was -0.60 (-0.81 - -0.39), -0.75 (-0.95 - -0.56) and -0.84 (-1.08 - -0.61) g/dL in the AL-placebo arm, the 0.25 mg/kg primaquine dose arm and the 0.40 primaquine dose arm, respectively (N=355, P=0.12).

Venous blood measurements

During follow-up, haematological parameters were also measured on venous blood samples collected on days 0, 3 and 7. When using haemoglobin levels measured on these venous samples to assess haemolysis, the average (95% CI) maximal fall in haemoglobin concentrations after treatment initiation was -0.19 (-0.30 - -0.07), -0.30 (-0.45 - -0.14) and -0.33 (-0.45 - -0.20) g/dL in the AL-placebo arm (N=110), the 0.25 mg/kg primaquine dose arm (N=122) and the 0.40 primaquine dose arm (N=119), respectively (P=0.32).

Reticulocytes

16 children had reticulocyte levels higher than 5% at enrolment. During follow-up, in 47 scheduled visits (17 on day 3 and 30 on day 7), reticulocyte levels were above 5%: 14, 18 and 15 in the AL-placebo, 0.25 mg/kg primaquine and 0.40 mg/kg primaquine arms, respectively. Median absolute reticulocyte count at enrolment was 88 (range 66 – 113) x10^3 reticulocytes per μL; absolute levels increased on day 7 versus day 0 in 78/101, 93/116 and 89/110 children in the AL-placebo, 0.25 mg/kg primaquine and 0.40 mg/kg primaquine arms, respectively. There was a positive correlation between increase in reticulocyte levels during the first week of follow-up and haemoglobin drop by day 7 in the primaquine arms (Spearman's rank correlation coefficient 0.18 [P=0.03], N=141).

Parasitological criterion

20 children with less than 1,000 asexual falciparum parasites per μL and no gametocytes at screening were mistakenly enrolled. They were equally distributed in the different study arms: 6, 7 and 7 were enrolled in the AL-placebo, the 0.25 mg/kg primaquine and the 0.40 mg/kg primaquine arms, respectively. 3, one from each study arm, of these 20 children had gametocytes on day 0 and were

included in the gametocyte clearance analysis. Additionally, one child, from the AL-placebo group, had *P. malariae* infection detected by microscopy at enrolment, but not during follow-up visits. Data from these children were included in analyses presented in this manuscript.

Tables

Table S1. Prevalence of microscopically-detectable gametocytes during follow-up in children with patent gametocytes on day 0. AL = artemether-lumefantrine; PQ = primaquine

	**AL**<br>**% (n/N)**	**AL + 0.25 mg/kg PQ**<br>**% (n/N)**	**AL + 0.40 mg/kg PQ**<br>**% (n/N)**
Day 2	38.6 (17/44)	35.4 (17/48)	36.5 (19/52)
Day 3	34.1 (15/44)	35.4 (17/48)	23.5 (12/51)
Day 7	20.9 (9/43)	10.4 (5/48)	5.8 (3/52)
Day 10	19.5 (8/41)	4.1 (2/49)	0.0 (0/51)
Day 14	15.4 (6/39)	0.0 (0/44)	0.0 (0/45)

Table S2. Gametocyte levels (per μL) measured by qRT-PCR during gametocyte-positive visits in children with patent gametocytes on day 0. AL = artemether-lumefantrine; PQ = primaquine

	**AL**<br>**Median (IQR, N)**	**AL + 0.25 mg/kg PQ**<br>**Median (IQR, N)**	**AL + 0.40 mg/kg PQ**<br>**Median (IQR, N)**	**AL vs. AL + 0.25mg/kg PQ**<br>**P-value**	**AL vs. AL + 0.40 mg/kg PQ**<br>**P-value**
Day 0	17.26 (7.21 – 45.08, 43)	18.62 (7.76 – 75.27, 48)	16.63 (7.18 – 39.99, 49)	0.58	0.83
Day 2	2.33 (0.81 – 10.21, 41)	2.25 (0.75 – 5.83, 42)	2.52 (0.85 – 9.70, 47)	0.57	0.97
Day 3	0.64 (0.08 – 2.64, 38)	0.87 (0.30 – 2.28, 37)	0.70 (0.17 – 2.49, 48)	0.35	0.67
Day 7	0.48 (0.12 – 1.28, 28)	0.14 (0.08 – 0.45, 17)	0.15 (0.05 – 0.24, 15)	0.12	0.02
Day 10	0.49 (0.17 – 1.40, 27)	0.12 (0.08 – 0.54, 14)	0.13 (0.05 – 0.31, 11)	0.07	0.008
Day 14	0.30 (0.18 – 0.50, 17)	0.10 (0.10 – 0.66, 5)	0.15 (0.12 – 0.53, 9)	0.56	0.77

Table S3. Adverse Events by treatment arm. In (A), adverse events (AEs) are presented by severity and causality; in (B), by clinical condition. AL = artemether-lumefantrine; PQ = primaquine

(A)

**Adverse Events by Severity and causality***	**AL only**	**AL + 0.25 mg/kg PQ**	**AL + 0.40 mg/kg PQ**
Any AE	15	18	24
Mild AEs	7	7	9
Mild AEs possibly related to study	2	1	4
Moderate AES	8	11	15
Moderate AEs possibly related to study	1	0	0
Severe AEs	0	0	0

*In total 48 children developed AEs during follow-up; 8 had 2 or more AEs.

(B)

**Adverse Event**	**AL only**	**AL + 0.25 mg/kg PQ**	**AL + 0.40 mg/kg PQ**
Bronchitis	2	4	6
Fever	2	4	5
Abdominal Pain	1	1	0
Vomiting[α]	2	2	2
Uncomplicated Malaria	2	1	2
Rhinitis/ Rhino-Bronchitis	1	1	1
Otitis	0	0	2
Dysentery	1	2	3
Epistaxis	0	0	1
Dental Pain	1	0	0
High transaminase levels	1	0	0
Palpebral inflammation	1	0	0
Muscle pain (Neck)	0	1	0
Wound	1	1	1
Trauma on left foot	0	1	0
Inflammation (left foot)	0	0	1

[α] 5/6 vomiting episodes occurred on days 0 or 1; for all these 5 visits, study drug was re-administered. 1 child vomited on day 3 after treatment initiation

Table S4. Maximal percentage drop in haemoglobin levels, relative to baseline (enrolment or screening), by treatment arm. AL = artemether-lumefantrine; PQ = primaquine.

	**Maximal % drop in haemoglobin**<br>**Mean % (95% Confidence Interval)**
AL only	5.7 (3.3 - 8.1)
AL + 0.25 mg/kg PQ	7.8 (5.8 - 9.8)
AL + 0.40 mg/kg PQ	9.9 (7.9 - 11.9)

Table S5. Number of participants with substantial haemoglobin drops (2 or more g/dL) during follow-up (N=35). AL = artemether-lumefantrine; PQ = primaquine.

	**Haemoglobin drop (in g/dL)**		
	2 - 3	3 – 4	> 4
AL only	6	3	0
AL + 0.25 mg/kg PQ	11	0	1
AL + 0.40 mg/kg PQ	9	4	1

Table S6. Laboratory abnormalities during follow-up (days 3 and 7). In addition to the findings presented in this table, 7 children presented white blood cell counts below 4,000 cells per µL during follow-up: 2, 3 and 2 in the AL-placebo arm, the 0.25 mg/kg primaquine dose arm and the 0.40 mg/kg primaquine dose arm, respectively. AL = artemether-lumefantrine; PQ = primaquine.

	**White blood cell counts > 15,000 / µL**
**Treatment arm**	**Number of children**
AL only	3
AL + 0.25 mg/kg PQ	2
AL + 0.40 mg/Kg PQ	1
	**Platelet count < 150,000 / µL**
**Treatment arm**	**Number of children**
AL only	4
AL + 0.25 mg/kg PQ	5
AL + 0.40 mg/Kg PQ	4
	**Creatinine levels above upper limit of normal range**[*,ϕ]
**Treatment arm**	**Number of children**
AL only	2
AL + 0.25 mg/kg PQ	1
AL + 0.40 mg/Kg PQ	1
	**Liver transaminases levels above upper limit of normal range**[¥,¤]
**Treatment arm**	**Number of children**
AL only	4
AL + 0.25 mg/kg PQ	2
AL + 0.40 mg/Kg PQ	1
	**Total bilirubin levels above 20 µmol/L**
**Treatment arm**	**Number of children**[α]
AL only	2
AL + 0.25 mg/kg PQ	5
AL + 0.40 mg/Kg PQ	7

** < 6 years: 44.2 µmol/L; 6 - 10 years: 53.0 µmol/L; 10-15 years: 61.9 µmol/L*

[ϕ] *2/4 had normal creatinine levels by the end of the follow-up*

[¥] *AST: < 6 years: 79 U/L; 6 - 10 years: 74 U/L; 10-15 years: 69 U/L*

ALT: < 6 years: 80 U/L; 6 - 10 years: 78 U/L; 10-15 years: 76 U/L

[¤] In 6/7 children with liver transaminases above normal range, levels decreased during the follow-up

[α] *3 children, one in each treatment arm, had bilirubin levels above 20 µmol/L at enrolment*

Table S7. Summary of previous studies assessing post-artemether lumefantrine transmission potential. Mosquito infection rates in the study by Ouédraogo and colleagues were based on PCR; the other studies listed in the table used microscopy to assess oocyst positivity.

	Study type	Setting	Inclusion trial	Gametocyte positivity required for feeding assay?	Number of assays	Time from treatment initiation (feeding assay)	Number of infected mosquitoes in feeding assay (AL only group)	Number of infectious individuals in feeding assay (AL only group)
Sutherland et al. 2005	RCT*	Gambia	Children with uncomplicated malaria	Yes	10	Day 7	0/196 (0)	0/10
Bousema et al. 2006	RCT	Kenya	Children with uncomplicated malaria	No	25	Day 14	27/750 (3.6%)	15/25
Sawa et al. 2013	RCT	Kenya	Children with uncomplicated malaria	No	77	Day 7	43/2293 (1.9%)	24/77
Ouédraogo et al. 2015	RCT	Burkina Faso	Asymptomatic adults	No**	68	Days 1 and 7	4/560 (0.7%)	-

* randomised controlled trial

** Although gametocyte positivity was not a criterion for inclusion in feeding assays, only children with positive Pfs25 mRNA NASBA results had mosquito infection status assessed

Sutherland CJ et al. Reduction of malaria transmission to Anopheles mosquitoes with a six-dose regimen of co-artemether. PLoS Med 2005, 2(4):e92

Bousema JT et al: Moderate effect of artemisinin-based combination therapy on transmission of Plasmodium falciparum. J Infect Dis 2006, 193(8):1151-1159

Sawa P et al. Malaria transmission after artemether-lumefantrine and dihydroartemisinin-piperaquine: a randomized trial. J Infect Dis 2013, 207(11):1637-1645

Ouédraogo et al. Efficacy and safety of the mosquitocidal drug ivermectin to prevent malaria transmission after treatment: a double-blind, randomized, clinical trial. Clin Infect Dis. 2015.
